# The Predicted Secretome of the Plant Pathogenic Fungus *Fusarium graminearum*: A Refined Comparative Analysis

**DOI:** 10.1371/journal.pone.0033731

**Published:** 2012-04-06

**Authors:** Neil A. Brown, John Antoniw, Kim E. Hammond-Kosack

**Affiliations:** Centre for Sustainable Pest and Disease Management, Department of Plant Pathology and Microbiology, Rothamsted Research, Harpenden, Hertfordshire, United Kingdom; University of Nebraska, United States of America

## Abstract

The fungus *Fusarium graminearum* forms an intimate association with the host species wheat whilst infecting the floral tissues at anthesis. During the prolonged latent period of infection, extracellular communication between live pathogen and host cells must occur, implying a role for secreted fungal proteins. The wheat cells in contact with fungal hyphae subsequently die and intracellular hyphal colonisation results in the development of visible disease symptoms. Since the original genome annotation analysis was done in 2007, which predicted the secretome using TargetP, the *F. graminearum* gene call has changed considerably through the combined efforts of the BROAD and MIPS institutes. As a result of the modifications to the genome and the recent findings that suggested a role for secreted proteins in virulence, the *F. graminearum* secretome was revisited. In the current study, a refined *F. graminearum* secretome was predicted by combining several bioinformatic approaches. This strategy increased the probability of identifying truly secreted proteins. A secretome of 574 proteins was predicted of which 99% was supported by transcriptional evidence. The function of the annotated and unannotated secreted proteins was explored. The potential role(s) of the annotated proteins including, putative enzymes, phytotoxins and antifungals are discussed. Characterisation of the unannotated proteins included the analysis of Pfam domains and features associated with known fungal effectors, for example, small size, cysteine-rich and containing internal amino acid repeats. A comprehensive comparative genomic analysis involving 57 fungal and oomycete genomes revealed that only a small number of the predicted *F. graminearum* secreted proteins can be considered to be either species or sequenced strain specific.

## Introduction

The intimacy of an association between a microbe and plant host is represented by foreign cells growing within plant tissue or even within living plant cells. Communication through the secretion of proteins and metabolites that are either taken up by the host or detected at the cell surface plays a pivotal role in determining the outcome of the interaction. Secreted proteins from animal-infecting malaria parasites and plant-infecting oomycete pathogens possess a conserved RxLR motif that facilitates protein secretion and uptake into the host cells resulting in the modulation of host transcription [1], [2], [3]. Fungi and oomycetes have convergently evolved a range of mechanisms to acquire nutrition from various habitats, including mutualistic, biotrophic, hemibiotrophic, necrotrophic and non-pathogenic saprophytic lifestyles. In fungi, no widely conserved translocation motif has been discovered, yet many small secreted proteins and metabolites are proven virulence factors [4]. However, a degenerative Y/F/WxC motif discovered in *Blumeria graminis* f. sp. *hordei* has been proposed to be conserved among intracellular non-necrotrophic ascomycetes [5]. Experimentally, secreted proteins termed ‘effectors’ that modulate the interaction between pathogenic microbes and hosts have been identified from all lifestyles. Examples include; the Avr and Ecp proteins from the tomato leaf mold fungus *Cladosporum fulvum* [6], the Tox proteins from the wheat glume blotch fungus *Stagonospora nodorum* [7], Avra10 and Avrk1 from the barley powdery mildew fungus *B. graminis* f. sp. *hordei* [8], the SIX proteins from the vascular wilt fungus *Fusarium oxysporum* f. sp. *lycopersici* [9], the Avr-Pita and Pwl proteins from the rice blast fungus *Magnaporthe oryzae* [10], Pep1 and Pit1/2 from the corn smut fungus *Ustilago maydis* [11], 3LysM from the wheat leaf blotch fungus *Mycosphaerella graminicola* [12] and Sp7 from the tomato mutualist *Glomus intraradices* [13].

Several apoplastic cysteine-rich and LysM containing fungal effectors have been shown to inhibit plant chitinases and/or bind chitin to prevent elicitation of pathogen associated molecular pattern (PAMP) triggered immunity (PTI) and thereby prevent the induction of host defences. These include the *C. fulvum* effectors Avr4/Ecp6 and *M. graminicola* 3LysM effector [12], [14], [15]. Recently the accelerated evolution of secreted proteins through internal amino acid repeats, which increase phenotypic plasticity, has been shown to influence the elicitation of the host's defence response [16], [17], [18]. Several intracellular effectors contribute to virulence in a different way. For example, Sp7 and Pwl2 are translocated to the host nucleus where they influence host transcription [13], [19]. Alternatively in *U. maydis*, Pep1 accumulates at the site of cell-to-cell passage and is essential to the establishment of infection [11], while clusters of effectors contribute to organ specificity [20]. Different again are the small necrotrophic effectors (Tox proteins) that induce host programmed cell death (PCD) to assist infection [7]. These examples from fungi with different *in planta* lifestyles demonstrate how different types of secreted fungal proteins define the outcome of an interaction between a microbe and its host.

Globally, the homothallic ascomycete fungus *Fusarium graminearum* (Teleomorph: *Gibberella zeae*) is the predominant causal agent of Fusarium Ear Blight (FEB) disease, also referred to as Head Scab (www.scabusa.org). This disease affects most small grain cereal species including wheat, barley and maize and has been associated with up to 17 *Fusarium* species. Serious and repeated FEB outbreaks have been reported in all major wheat producing countries (www.faostat.fao.org) and consequently, the international maize and wheat improvement centre (CIMMYT) describes FEB as a major limiting factor to production [21]. This re-emergence is thought to be driven by changes in agronomic practices as well as to climatic changes. In addition to reducing grain yield and product quality, the crop is also contaminated with mycotoxins that are harmful to both animals and humans [22]. Due to the health threat, farmers in the EU and USA pay for their grain to be tested for the presence of the type B trichothecene mycotoxin deoxynivalenol (DON) and to determine that the levels are below the recommended safety guidelines (www.hgca.com, wwww.USDA.com) [23]. It is estimated that one infected ear per square metre of the wheat crop is sufficient for the DON concentrations in the grain to exceed safe levels.

*F. graminearum* was one of the first plant pathogens to be selected for full genome sequencing due to the growing global importance of the disease, the large number of cereal species infected and the growing health concerns. The sequenced *F. graminearum* genome of the PH-1 strain was found to be 36.1 Mb in size and due to the availability of a genetic map between the PH-1 and a second strain of USA origin (MN00-676), this genomic sequence was immediately aligned to the four chromosomes [24]. Due to the homothallic nature of *F*. *graminearum* and because an active repeat-induced point mutation system operates during each meiosis, the genome contains little repetitive DNA and no active transposable elements compared to related fungi [25]. The latest version of *F. graminearum* genome available from MIPS (version FG3.2) has a considerable amount of manual annotation incorporated and is predicted to encode 13,718 genes [26]. In the original genome analysis of ∼11,600 genes [25], the TargetP defined secretome was predicted to account for approximately one tenth (1,442) of the predicted genes. Low level sequence coverage of a second *F. graminearum* strain, GZ3639, demonstrated the non-random distribution of nucleotide polymorphism in the genome, with hot spots of sequence variation occurring in sub-telomeric and central regions [25]. These highly variable ‘hot’ regions of the genome were found to be enriched for genes coding for predicted secreted proteins. An analysis of the genomic location of experimentally proven *F. graminearum* pathogenicity/virulence genes and homologues of verified pathogenicity/virulence genes from other species (www.PHIbase.org) has revealed that most genes with this function resided in regions of low level recombination [27]. Their location in the ‘cooler’ parts of the genome has been suggested to protect them from gene loss [28]. The majority of these genes code for conserved intracellular proteins involved in signal transduction, such as the mitogen activated protein kinases (MAPKs), and represent ancient conserved signalling pathways recruited by pathogens to co-ordinate infection [29]. Within *F. graminearum* genomic regions found to exhibit high recombination frequencies [25] reside genes that encode for small and large sized secreted proteins. For instance, an abundance of plant cell wall degrading enzymes (PCWDEs) was identified. This type of genome positioning is hypothesised to assist the evolution of the pathogen in the rapidly changing arms race with its host. By contrast, in the oomycete *Phytophthora infestans* the vast majority of the predicted secretome, which is evolving rapidly, is located in regions of the genome where an abundance of transposon sequences reside [30]. A similar genome location, rich in transposon sequences, is now recognised to harbour the predicted secretome of the Ascomycete powdery mildew fungus, *B. graminis* f. sp. *hordei* [31].

Production of the water soluble, secreted trichothecene mycotoxin, deoxynivalenol (DON), is required by *F. graminearum* for full virulence on wheat ears, but not for full virulence on barley ears, maize cobs or the floral tissue of the model species *Arabidopsis thaliana* [32], [33], [34], [35], [36]. DON inhibits protein synthesis in eukaryotes and prevents polypeptide chain initiation or elongation by binding to the 60S ribosomal subunit [37]. In *F. graminearum* infections of wheat, the trichothecene mycotoxin genes within the *Tri* cluster are most highly expressed during symptomless infection [38]. Wheat ear infection by the non-DON producing *tri5* gene deficient mutant results in an enhanced defence response in the form of plant cell wall thickening adjacent to the invading *Fusarium* hyphae [39]. In the absence of DON production, the interaction between the two organisms at the infection front is altered. A macroscopically visible brown ring forms around the slowly expanding lesion on the glumes of wheat ears sprayed with *F. graminearum* [34]. Topoisomerase modulation of DNA topology has been demonstrated to regulate virulence gene expression, especially secreted proteins [40], [41]. The *top1* deficient *F. graminearum* strain was unable to colonise the wheat ear despite producing wild-type DON levels and infections were restricted to just below the surface of the floral brackets [42]. While the secreted lipase *fgl1-*deficient strain produced enhanced DON *in planta*, yet in wheat ears an extensive host cell browning reaction was evident in the tissue immediately beyond the confined *Fusarium* hyphae [43]. Collectively this implies that additional virulence factors, in combination with the secreted DON mycotoxin, promote symptomless infection and implicates a role for secreted proteins in *F. graminearum* pathogenicity.

In view of these findings and the recently identified symptomless phase of wheat ear infection where the *F. graminearum* hyphae advance exclusively extracellularly between the wheat cells [44], we decided to explore in detail the predicted secretome. This new study of the secretome could potentially give the first clues to what proteins are involved in the establishment/maintenance of symptomless infection, as well as the transition from extracellular to intracellular growth. Various bioinformatic tools that assist the prediction of fungal secretomes are available. These tools utilise different but highly complementary analytical approaches, namely the prediction of the presence of a signal peptide (SignalP/TargetP) and predicting the eventual cellular location of the mature protein (WolfPSort). Used individually these approaches often predict non-secreted proteins as secreted, but when used in combination an increased accuracy of the prediction was anticipated. In this study we describe, in detail, a refined prediction and possible function of the *F. graminearum* secretome. In addition, a genomic comparison of the *F. graminearum* secretome with 57 similarly predicted fungal and oomycete proteomes, including other Fusaria and many pathogenic/non-pathogenic species, has been used to partition this predicted secretome into species specific, genera specific and highly conserved gene sets.

## Materials and Methods

### Bioinformatic analyses of the secretome

The FG3 version of the genome was downloaded from MIPS (http://mips.helmholtz-muenchen.de/genre/proj/FGDB/) in October 2009. The prediction of the refined *F. graminearum* secretome was based on the procedure described by Muller and colleagues [45] for *U. maydis*. We developed an automated secretome prediction pipeline based on this procedure using bash shell, AWK and Python scripts on a PC running Red Hat Linux 5.2. Initially all proteins with a Target P Loc = S (TargetP v1.1; http://www.cbs.dtu.dk/cgi-bin/nph-sw_request?targetp) and a Signal P D-score = Y (SignalP v3.0; http://www.cbs.dtu.dk/cgi-bin/nph-sw_request?signalp) were combined [46], [47]. These were then scanned for transmembrane spanning regions using TMHMM (TMHMM v2.0; http://www.cbs.dtu.dk/cgi-bin/nph-sw_request?tmhmm) and all proteins with 0 TMs or 1 TM, if located in the predicted N-terminal signal peptide, were kept. GPI-anchor proteins were predicted by big-PI (http://mendel.imp.ac.at/gpi/cgi-bin/gpi_pred_fungi.cgi) [48]. ProtComp was also used to predict localization of the remaining proteins using the LocDB and PotLocDB databases (ProtComp v8.0; http://www.softberry.com). All proteins predicted as extracellular or unknown were kept in the final secretome dataset. Pfam analysis was done using the Pfam database (ftp://ftp.ncbi.nih.gov/pub/mmdb/cdd/) and the rpsblast program in the NCBI blast+ software package (ftp://ftp.ncbi.nlm.nih.gov/blast/executables/blast/). WolfPSort analysis was done using “runWolfPsortSummary fungi” in the WoLFPSORT v0.2 package [49]. The number of cysteine residues within the mature peptide and the search for degenerative Y/F/WxC motifs were computed using custom Python scripts. The number of internal amino acid repeats was found using RADAR (http://www.ebi.ac.uk/Tools/Radar/) [50]. The detection of RNA transcripts for the 574 *F. graminearum* genes of interest was explored using Affymetrix gene expression data generated in several published *in planta* and *in vitro* investigations (Experiments FG1, FG2, FG15 and FG16) downloaded from www.PLEXdb.org.

### Analysis of chromosome location alongside other key features of the *F. graminearum* genome

To inspect the position of individual or clusters of genes on the four *F. graminearum* chromosomes, the Fgra3Map tool was downloaded from www.Omnimapfree.org which displays a map of the complete *F. graminearum* genome (MIPS version 3.1). The Fgra3Map was used according to methods described [51].

### Comparative analysis of the refined *F. graminearum* secretome

For the detailed follow up analyses, only proteins with a predicted signal peptide sequence and a value of extr 18 or greater from the WolfPSort analysis were used. The *F. graminearum* secretome was compared with 57 other fungal and oomycete genomes of pathogens varying in host range, tissue specificity and lifestyle as well as several exclusively saprophytic species (Table S1). The fungal and oomycete genomes and their predicted gene repertoires were downloaded from either the BROAD or JGI websites or from species specific websites maintained by various research communities. For the comparative analyses, the conservation, absence or expansion of the genes coding for the *F. graminearum* secreted proteins was explored by BLASTP analysis, determined at two levels of confidence, p<e^−5^ and p<e^−40^.

## Results

### The secretome of *F. graminearum*

**Figure 1:**
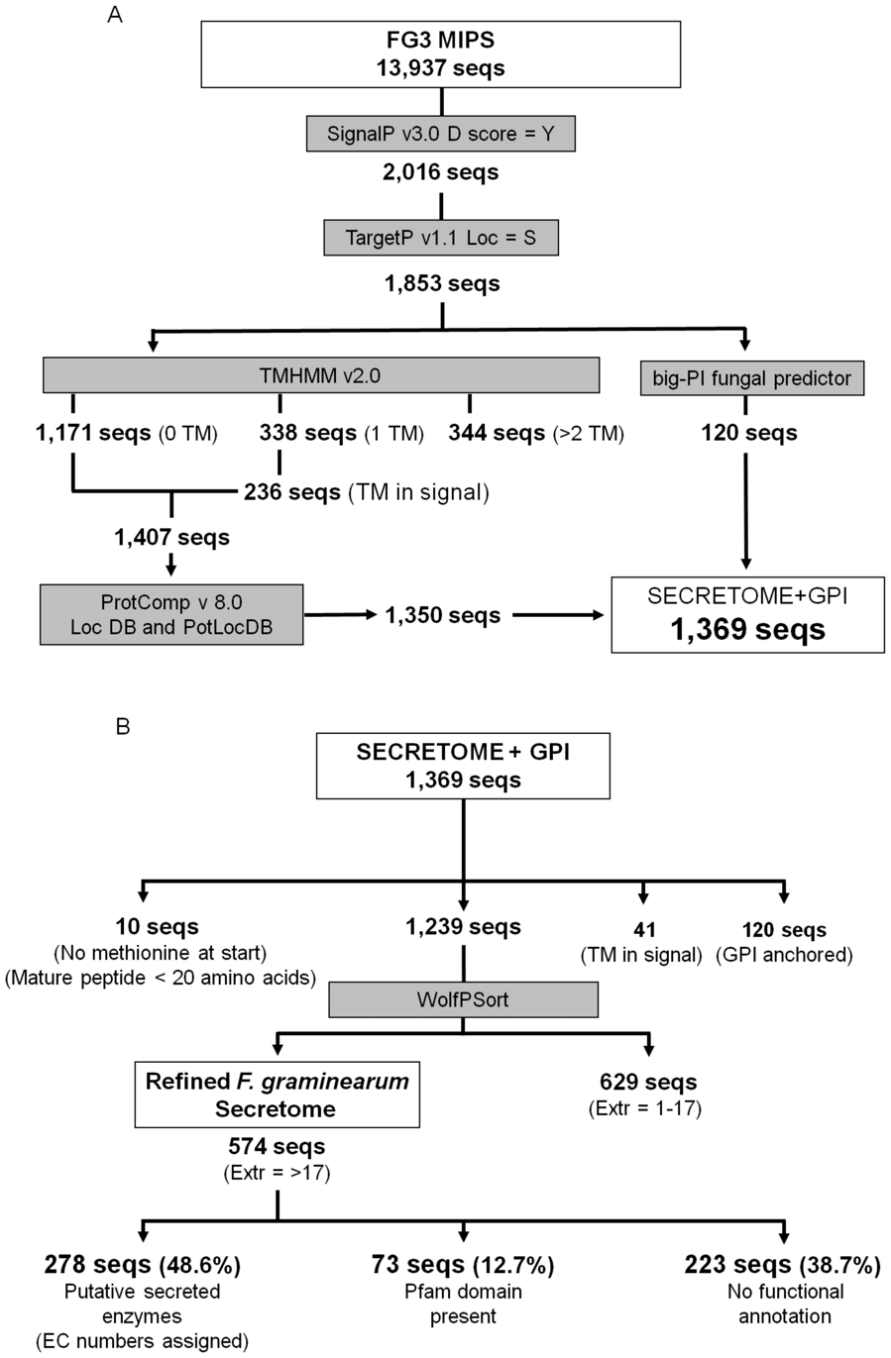
The bioinformatics pipelines used to predict the *F. graminearum* secretome. (**A**) The total secretome and (**B**) the refine secretome.

In the original genome paper [25], the secretome was predicted from the FG1 gene call using only the TargetP software. In the current study we analysed an updated, refined FG3 gene call (13,937 proteins) of the *F. graminearum* genome in two phases. In the first phase (Figure 1A), designed to predict all possible secreted proteins, SignalP and TargetP were used to identify secreted proteins with signal peptides (1,853 proteins) and those predicted to contain GPI anchors (120 proteins) were identified. After removal of the signal peptide, any mature proteins that contained a transmembrane domain were excluded. An initial screen used ProtComp software to exclude proteins that were probably not located in the extracellular space. This produced a set of 1,369 secreted proteins (including those with GPI anchors). Phase 2 (Figure 1B), designed to identify proteins with a high probability of being secreted, contained more stringent conditions to further refine this set of proteins, discarding both those that did not begin with a methionine and small proteins where the mature proteins were shorter than 20 amino acids. At this stage, the 41 proteins with a TM domain predicted within the signal peptide sequence were also excluded. Similarly, all proteins predicted to contain a GPI-anchor were removed. A second software package (WolfPSort) that predicts the eventual location of proteins was used to only identify proteins that are secreted into the extracellular spaces (extracellular score >17). This resulted in a reduced set of 574 secreted proteins. In total 99% of the refined *F. graminearum* secretome is supported by transcriptional evidence from published *in vitro* and *in planta* investigations. Five of the fungal genes included within the refined secretome have not been assigned Affymetrix probe-sets and are therefore not supported by transcriptional evidence.

For completeness, and to assist with follow up comparative analyses, the results for the predicted secretome with the larger size of 1,369 genes arising from phase A of the analysis are presented in Spreadsheet S1.

The MIPS annotation and functional classification was determined for the 574 secreted proteins present in the FG3 gene call (http://mips.helmholtz-muenchen.de/genre/proj/FGDB/). Of these, 278 proteins possessed information on protein function whilst 296 proteins were described as hypothetical or conserved hypothetical.

**Figure 2:**
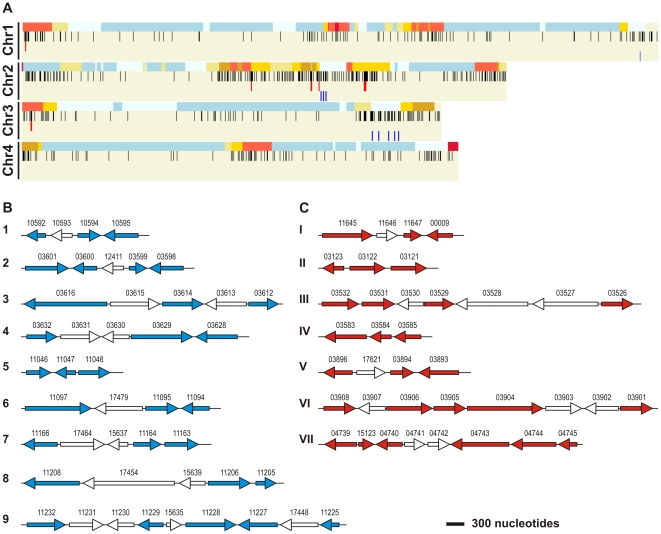
The macro- and micro-chromosome locations of the genes predicted to encode the refined *F. graminearum* secretome. (**A**) Gene distribution across the four chromosome (Chr 1–4), where each black vertical bars represents a single gene (n = 574), aligned next to a heat map for genetic recombination (red = high to blue = low, recombination frequency - upper row of each chromosome) displayed on Fgra3Map. Some *F. graminearum* genes that encode secreted proteins were organised in clusters. The secretome was divided into genes that reside in regions of low (blue bars) or high frequency recombination (red bars) and displayed on Fgra3Map. Details of the gene clusters coding for secreted proteins in low (**B**) and (**C**) high recombination regions. Clusters are presented in chromosome order, while the coloured arrows (secreted proteins) or white arrows (non-secreted proteins) represent gene orientation. Arrow length is proportional to gene length with the length of the scale bar representing 300 nucleotides. Genes are labelled with their respective FGSG identifiers.

The chromosomal location of the genes encoding the 574 secreted proteins was compared to the recombination frequency across the four chromosomes using Fgra3Map software. Genes coding for secreted proteins were identified on all four chromosomes and were found to be preferentially located within sub-telomeric regions and regions with a high recombination frequency (Figure 2). A similar distribution pattern had been noted in the original FG1 analysis [25]. Within this overall pattern, the annotated genes and the unannotated genes present in the refined secretome were equally represented in the high and low recombination regions of the genome. To inspect whether any *F. graminearum* genes that encode secreted proteins were organised in clusters the secretome was divided into genes that reside in regions of low or high recombination frequency and displayed on the genome. A few small clusters that demonstrated no clear conservation in function were identified in regions of low and high recombination (Figure 2). In total, 51.6% of these genes were annotated as either hypothetical or conserved hypothetical. Secretome clusters were small in size, containing three to nine genes and were coded for by either DNA strand. The clusters within areas of high recombination were sub-telomerically located on chromosomes 1 and 3 as well as an interstitial hot spot on chromosome 2. Eight clusters resided in regions of low recombination and were closely located within a 97 and 495 Kb region of chromosomes 2 and 3, respectively. The 9^th^ cluster was located in a ‘cool’ sub-telomeric region on chromosome 1. No clusters were found on chromosome 4.

### Analysis of the proteins with a predicted function

Closer inspection of the 278 proteins possessing information on protein function revealed that 243 contained at least one Pfam domain. A sub-set of 171 proteins was predicted to be involved in the degradation of plant derived compounds. These were divided according to substrate specificity (Table 1, Tables S2 and S3). After excluding five fungal chitinases, the remaining 102 annotated proteins considered not to be involved in plant substrate degradation were organised according to their MIPS functional category. Each sub-set is described in turn.

**Table 1:** The number of secreted *F. graminearum* proteins possibly involved in the degradation of the different components of the wheat host cell.

Plant cell component	Target for degradation	Number of secreted proteins
Waxy cuticle	Cuticle	2
Plant cell wall	Cellulose	30
	Hemicellulose	46
	Lignin	9
	Callose	9
Plant cell wall and the middle lamella	Pectin	13
Plasma membranes and fat bodies	Lipids	19
Starch bodies	Starch	3
Situated throughout the cell, e.g. wall, membrane and protein bodies	Proteins	37
Plasma membranes	Choline	3
**Total**		**171**

Almost all host plant surfaces are coated by a waxy cuticle, which represents the first barrier to plant infection. The plant cell wall beneath consists of cellulose microfibrils cross- linked by an amorphous matrix of hemicellulose and pectin, often encased in lignin polymers as the plant matures. The *F. graminearum* secretome possesses an arsenal of secreted proteins and enzymes that target the plant cuticle and each of the cell wall components. This arsenal potentially involving up to 109 secreted proteins (Table 1 and Table S2). Thirty secreted proteins involved in the degradation of cellulose were identified, four of which were predicted to bind cellulose, fourteen were predicted to target β-1,4 glucans and twelve to target the breakdown product cellobiose. Enzymes that modify the different polysaccharides which make up hemicellulose represent the largest group of secreted cell wall modifying proteins and this reflects the diversity in hemicellulose composition. The two major components of hemicelluloses, arabinose and xylan, were targeted by the greatest number of secreted proteins. Nine secreted enzymes were detected that degrade the phenolic polymer lignin and its crosslinks to hemicellulose, including laccases, peroxidases and ferulic acid esterases. Multiple pectate lyases and pectin esterases were found (n = 13) that breakdown pectin in the middle lamella and cell wall of the plant. Callose is a polysaccharide of β-1,3 glucan that exists in plasmodesmata, phloem sieve plates and is laid down in response to wounding or imminent pathogen attack. Nine enzymes that target callose were identified, including endo- and exo- β-1,3 glucosidases.

Beyond the plant surface and the cell wall, the rest of the plant cell consists of proteins, lipid, sugars and nucleic acids. In total, 37 protein digesting enzymes were identified (Table 1 and Table S3), and included multiple alkaline/neutral and serine/aspartyl proteinases as well as amino, carboxy and endo peptidase. In contrast, only three enzymes were identified that were predicted to breakdown starch into sugars suitable for uptake by the fungal cell. These were two amylases and a glucose dehydrogenase. A high number of secreted enzymes that target lipids were identified, including 15 triacylglycerol lipases. Therefore, *F. graminearum* secretes an array of proteins that possess the ability to degrade and utilise the plant cell in its entirety. In 2006, a comparative genome analysis including *Ustilago maydis* and various newly available completed fungal genomes, before the *F. graminearum* genome was published [25], predicted the *F. graminearum* genome to encode 103 plant cell wall degrading enzymes [52].

**Figure 3:**
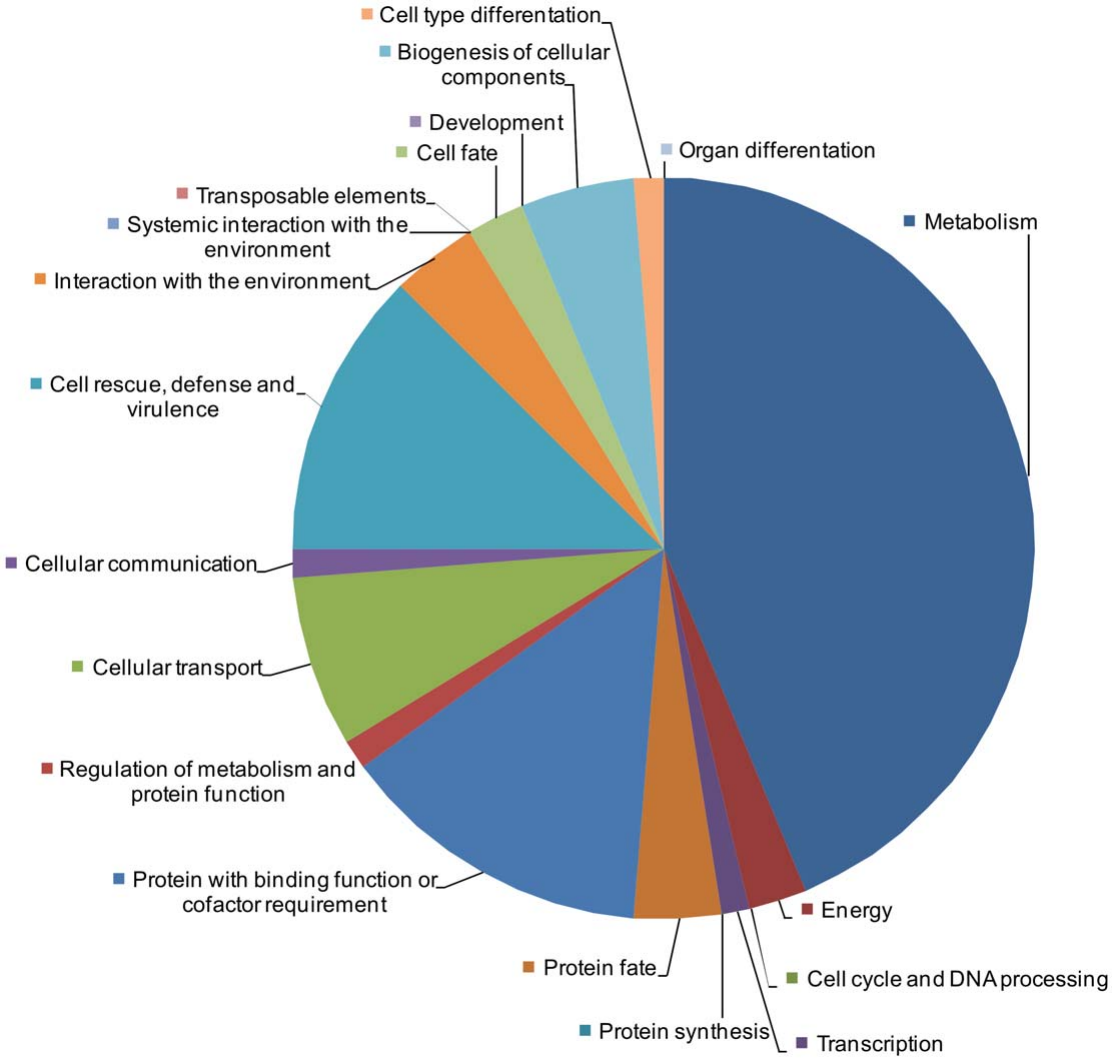
The sub-set of MIPS annotated secreted *F. graminearum* proteins, which are predicted not to be involved in the degradation of plant cells. The proteins were organised according to their MIPS functional category (http://mips.helmholtz-muenchen.de/genre/proj/FGDB/). N = 102.

The sub-set of 102 MIPS annotated secreted proteins not predicted to function in the degradation of plant cells were organised according to their MIPS functional category and scrutinised further (Table S4). Within this diverse selection of proteins, those involved in metabolism accounted for the greatest proportion (43%; Figure 3). These included acid/alkaline phosphatases, alcohol oxidases, a salicylate hydroxylase and four extracellular nucleases (Table 2).

**Table 2:** A selection of MIPS annotated secreted *F. graminearum* proteins, which are not involved in the degradation of plant cells, but are associated with metabolism or pathogenicity.

Function	FGSG_ID	MIPS Annotation
Acid/alkaline phosphatases	FGSG_04504	Related to acid phosphatase precursor
	FGSG_05933	Related to acid phosphatase precursor
	FGSG_06610	Related to alkaline phosphatase D precursor
	FGSG_07608	Related to acid phosphatase precursor
	FGSG_07678	Related to acid phosphatase Pho610
Salicylate hydroxylase	FGSG_08116	Related to salicylate hydroxylase
Extracellular nucleases	FGSG_02686	Related to ribonucleases
	FGSG_03379	Related to ribonucleases
	FGSG_11190	Probable ribonuclease T1
	FGSG_15003	Related to dnase1 protein
Phospholipases	FGSG_08150	Related to PLB1 - phospholipase B (lysophospholipase)
	FGSG_11236	Related to non-hemolytic phospholipase C precursor
PR-like proteins	FGSG_03109	Related to plant PR-1 class of pathogen related proteins
	FGSG_08549	Related to pathogenesis-related protein PR5K (thaumatin family)
Antifungal proteins	FGSG_00060	Related to KP4 killer toxin
	FGSG_00061	Related to KP4 killer toxin
	FGSG_00062	Related to KP4 killer toxin
	FGSG_04745	Related to antifungal protein
Pathogenicity related	FGSG_00006	Related to gEgh 16 protein
	FGSG_09353	Related to gEgh 16 protein
Phytotoxin	FGSG_10212	Probable SnodProt1 precursor
	FGSG_11205	Probable SnodProt1 precursor
Detoxification	FGSG_03816	Probable lactonohydrolase
	FGSG_10675	Related to lactonohydrolase

The other MIPS functional categories that were highly represented within the annotated sub-set were proteins with binding functions or cofactor requirements and cell rescue, defence and virulence. Two fungal proteins similar to plant pathogenesis related (PR) proteins PR1 in *Nicotiana tabacum* and PR5K pathogenesis-related thaumatin family protein in *A. thaliana* were identified. The only protein to possess both nuclear export and localisation signals was FGSG_04685. This protein contained a dioxygenase domain that incorporates O_2_ into an unknown substrate. Two *F. graminearum* secreted proteins were predicted to be phytotoxic and were highly related (6e^−40^ and 9e^−47^) to the *S. nodorum* phytotoxin, Snodprot1 [53]. Four other *F. graminearum* secreted proteins were predicted to possess antifungal properties and three of these were related to the KP4 killer toxin from *U. maydis* [54]. Two detoxifying lactonohydrolases were also identified.

Despite the stringency of the requisites of the predicted *F. graminearum* secretome several proteins believed to be intracellular were present. Two orthologs of *GEGH16* from the powdery mildew fungi *B. graminis* f. sp. *hordei* and Gas1 and Gas2 from *M. oryzae* that function in pathogenicity and penetration [55] were predicted to be secreted. However, fluorescently labelled Gas proteins localised to the cytosol of *M. oryzae* appressoria implying that the two *F. graminearum GEGH16* homologues may not be secreted [55]. The trichothecene 3-O esterase code by *TRI8* (FGSG_03532), was also predicted to be secreted, but this biosynthetic enzyme is not detected in culture filtrates of *F. sporotrichioides* [56] suggesting that it is also not extracellular in *F. graminearum*.

### Analysis of the proteins with no predicted function

The remaining 295 predicted secreted proteins that lacked annotation were analysed for internal amino acid repeats, high cysteine content and Pfam domains. The majority of these proteins, 190, are conserved. Protein functional domains can be predicted by sequence similarity. Using the Pfam database (http://pfam.sanger.ac.uk/) a total of 82 functional protein domains were found to be present within 73 secreted proteins, including 11 proteins with multiple Pfam domains (Table S5). The most abundant domain is Pfam04616 that belongs to the glycoside hydrolase 43 family (n = 5), which have been reported to have arabinofuranosidases, arabinanase and xylosidase activity. Other common domains included; a GDSL-like lipase domain (Pfam00657) involved in lipid metabolism (n = 4), a beta-lactamase domain (Pfam00144) associated with antibiotic resistance (n = 4), a nuclease/phosphatase family domain (Pfam03372) involved in intracellular signalling (n = 3), a necrosis inducing protein domain (Pfam05630) similar to the NPP1 protein from *P. infestans* [57] (n = 3), and a carbohydrate-binding domain (Pfam10528) found in fungal adhesins (n = 3).

The unannotated *F. graminearum* secreted proteins were screened at two thresholds, where the total number of cysteine residues represented greater than 5 or 10% of the mature protein. The majority (80%) of these predicted proteins had an even number of cysteine residues (Cys). At the >5% Cys threshold, 61 proteins were identified (31 conserved and 30 hypothetical proteins) (Table S6), whilst at the >10% Cys threshold, 11 proteins were identified (7 conserved and 4 hypothetical proteins) (Table 3). Four of these FGSG genes were not identified in the other *Fusarium* species (Table 3). Orthologs of the 29 *F. graminearum* proteins with 4, 6 or 8 cysteine residues were determined using BLASTP. Of these *F. graminearum* proteins 20 were conserved amongst the Fusaria while seven had no strong hits. Eight, five and three proteins were conserved in other saprophytes (*Aspergillus* species and *Neurospora crassa*), necrotrophs (*Botrytis cinerea*, *Cochliobolus heterostrophus*, *Sclerotinia sclerotiorum*, *S. nodorum* and *Pyrenophora tritici-repentis*) and hemibiotrophs (*M. oryzae*, *P. infestans* and *Verticillium albo-atrum*), respectively. The locus FGSG_03969 was atypical, coding for a somewhat larger mature protein at 482 amino acids in length of which 58 (12%) were cysteine residues and also contained 13 internal amino acid repeats.

**Table 3:** Analysis of *F.graminearum* secretome proteins and genes with no predicted function for cysteine content, the presence of internal amino acid repeats, Pfam domains, gene family size and presence in other Fusaria species and *Mycosphaerella graminicola*.

Locus_ID	MPL*	NC	%MP	RR	Pfam	Twin e-40	*Fv*	*Fol*	*Fs*	*Mg*
FGSG_00260	60	6	10	-	-	0	1.11e-28	-	5.32e-23	9.8e-20
FGSG_03969	482	58	12.03	13	-	0	4.14e-141	5.25e-158	8.65e-106	2.31e-97
FGSG_03599	77	10	12.99	3	-	0	3.93e-21	5.20e-27	-	-
FGSG_06712	129	16	12.4	3	-	0	1.12e-27	3.01e-27	5.56e-28	6.08e-14
FGSG_09066	64	8	12.5	2	-	0	4.00e-14	6.05e-15	1.56e-12	1.07e-22
FGSG_12214	79	10	12.66	2	-	0	5.23e-47	2.37e-47	-	-
FGSG_15142	71	8	11.27	-	-	0	-	-	-	-
FGSG_15437	53	8	15.09	-	-	0	-	-	-	-
FGSG_15448	71	8	11.27	-	-	0	-	-	-	0.00022
FGSG_15251	47	6	12.77	-	-	0	-	-	-	-
FGSG_15661	77	10	12.99	-	-	0	1.04e-21	-	-	-
FGSG_00002	714	70	9.8	15	-	1	9.94e-46	2.01e-129	8.05e-83	4.47e-95
FGSG_00031	1349	25	1.85	12	05109	1	-	-	1.22e-10	-
FGSG_00411	518	3	0.58	8	-	0	1.07e-85	2.08e-115	3.20e-46	6.03e-50
FGSG_00987	321	0	0	5	-	0	4.32e-40	3.22e-44	3.75e-12	-
FGSG_01570	363	4	1.1	6	-	0	2.68e-86	6.27e-92	5.31e-64	1.35e-60
FGSG_01588	708	18	2.54	5	09770	0	4.81e-59	2.12e-70	1.64e-34	2.88e-33
FGSG_02448	588	5	0.85	6	05109	0	3.69e-41	1.03e-50	1.35e-23	4.54e-22
FGSG_02888	997	41	4.11	5	05109	1	6.68e-78	1.14e-80	1.14e-41	5.87e-40
FGSG_02898	1195	98	8.2	10	-	0	1.50e-77	1.54e-47	-	6.52e-28
FGSG_03054	365	2	0.55	6	-	0	-	-	2.33e-165	7.02e-163
FGSG_03274	861	16	1.86	6	-	1	9.78e-143	2.73e-169	8.83e-07	8.26e-15
FGSG_04429	974	52	5.34	6	-	0	-	-	3.43e-28	5.26e-21
FGSG_04563	297	0	0	5	-	0	5.22e-10	-	-	-
FGSG_04824	682	9	1.32	9	09770	0	4.10e-23	3.39e-06	1.73e-06	1.45e-05
FGSG_04900	428	9	2.1	6	-	0	2.95e-45	2.86e-07	1.13e-32	1.38e-22
FGSG_05719	1136	20	1.76	6	-	1	-	-	-	3.79e-51
FGSG_06479	745	32	4.3	7	-	3	1.55e-164	1.75e-137	1.07e-80	4.51e-67
FGSG_09142	328	0	0	6	-	0	1.03e-164	1.11e-165	6.35e-131	6.76e-126
FGSG_10435	1746	40	2.29	9	M	0	-	-	-	-
FGSG_10676	995	4	0.4	17	**	2	9.35e-159	-	1e-45	1.53e-46
FGSG_10972	355	5	1.41	6	-	0	3.13e-08	9.90e-33	-	-
FGSG_11238	578	25	4.33	5	-	2	8.73e-60	7.18e-47	2.13e-111	1.54e-118
FGSG_11379	442	9	2.04	8	-	0	8.15e-52	9.56e-52	9.43e-52	8.44e-50
FGSG_12918	499	24	4.81	6	-	2	3.25e-106	5.08e-130	1.27e-70	9.21e-88
FGSG_12439	638	58	9.09	12	-	0	5.03e-58	4.75e-174	4.89e-95	6.05e-88
FGSG_13583	1862	0	0	25	-	0	-	-	-	2.49e-12
FGSG_00111	113	5	4.42	-	-	0	-	1.53e-52	-	-
FGSG_07755	86	6	6.98	-	-	0	1.43e-36	2.17e-49	2.76e-24	1.30e-21
FGSG_08026	754	14	1.86	4	-	2	3.39e-63	1.01e-60	2.64e-38	1.99e-47
FGSG_08213	328	10	3.05	2	-	1	-	9.73e-31	7.80e-11	3.93e-08
FGSG_09071	359	10	2.79	3	-	0	2.28e-06	3.48e-09	-	-
FGSG_11276	618	9	1.46	4	-	1	2.05e-47	1.28e-48	6.21e-21	5.66e-21
FGSG_11675	259	3	1.16	3	-	0	5.69e-48	2.79e-49	-	-
FGSG_12434	564	24	4.26	4	-	0	4.47e-54	1.44e-118	7.50e-158	2.28e-144
FGSG_12622	275	7	2.55	3	-	0	-	-	1.68e-08	4.35e-06
FGSG_13443	114	4	3.51	-	-	0	-	7.13e-43	-	-

* Abbreviations used in this table MPL = Mature peptide length, NC = Number of cysteine residues, Percentage cysteine residues in mature peptide, RR = Number of radar repeats, Pfam = Pfam domains, *Fv* = *F. verticillioides*, *Fol = Fusarium oxysporum* f. sp. *lycopersici*, *Fs* = *F. solani*, *Mg* = *Mycosphaerella graminicola*.

** Pfams: 05792/10528.

M = FGSG_10435 Pfams: 01034, 01822, 03154, 03935, 03999, 04415, 04484, 04683, 05109, 05110, 05539, 05642, 05792, 05955, 06075, 06933, 07010, 07218, 07263, 08550, 08580, 08601, 08639, 08702, 08729, 09319, 09595, 09726, 09786 and 10033.

All 574 sequences were inspected for the presence of the degenerative RxLR-dEER [1] and Y/F/WxC motifs [5] in close proximity to the predicted signal peptide sequence. No exact RxLR-dEER matches were found within the refined *F. graminearum* secretome. By contrast, a YxC motif was present in close proximity to the predicted signal peptide cleavage site in five proteins. These were three conserved hypothetical proteins (FGSG_00260, FGSG_01815 and FGSG_03050), an endoglucanase (FGSG_02658) and a hypothetical protein (FGSG_13505). The unannotated portion of the predicted secreted proteins were screened for the presence of both perfect and imperfect internal repeats using the RADAR software [50]. This analysis identified 28 proteins ranging from 297–1862 amino acids in length of which five were also cysteine-rich (>5%). Multiple copies of nine of these proteins were found by BLASTP at two different thresholds, four at e^−100^ and nine at e^−40^ (Table 3). The 28 secreted proteins of *F. graminearum*, which were predicted to contain internal repeats, were highly conserved. For example; *F. oxysporum* f. sp. *lycopersici* possessed 19, *F. solani* 21, *F. verticillioides* 22 and *M. graminicola* 22 orthologous proteins (Table 3).

Gene family size for each *F. graminearum* encoded secreted protein of unknown function was determined by BLASTP. As anticipated, the majority of the *F. graminearum* gene families were larger in the other *Fusarium* species (*F. oxysporum* f. sp. *lycopersici*, *F. solani* and *F. verticillioides*). A limited number of proteins including four conserved hypothetical and six hypothetical proteins demonstrated gene expansion within the Fusaria (Table 3). The function of these secreted proteins is unclear as they possess no Pfam domains. The *F. graminearum* secreted proteins were also screened for the expansion of gene families in other fungal organisms. A single gene, FGSG_08958, was expanded in *F. oxysporum* (43 copies) and *F. solani* (23 copies) as well as other saprophytic and soil dwelling organisms including 26 to 52 copies in *Trichoderma spp.*, 30 copies in *A. nidulans*, and 22 copies in *Chaetomium globosum*. The conserved hypothetical, FGSG_08958, contained a nucleoside phosphorylase domain. Four genes were dramatically expanded in *Phytophthora* species with up to 19 copies identified (p<e^−40^). Ten copies of a single gene FGSG_ 03708 were detected in *M. graminicola* and *M. fijiensis* (p<e^−40^) (Spreadsheet S1).

### Comparison of the predicted *F. graminearum* secretome with a broad range of fungal and oomycete species

A total of 57 genomes covering animal/plant pathogens, saprophytes and free living eukaryotic microbes were assembled. This list included 44 fungal and oomycete species (Table S1). The objective of this part of the study was to identify the *F. graminearum* specific secreted proteins, and the level of gene sequence conservation between species with a range of lifestyles or tissue specificities. Each genome was screened for the presence of *F. graminearum* secretome homologues (Spreadsheet S1). The results from this BLASTP analysis are reported at two levels of stringency, however for clarity the results obtained at a p value<e^−5^ are focused upon below.

The majority of the *F. graminearum* secretome was detected in all four *Fusarium* species assessed (78.05%), while 5.4% of the secretome was unique to these four *Fusarium* species (Table 4) including, 22 hypothetical proteins, eight conserved hypothetical proteins and a related cell wall mannoprotein. An additional 3.31% of the secretome was only found in *F. graminearum* (Table 4). These 19 genes that all encoded hypothetical proteins therefore represent either the species specific and/or strain specific secreted gene repertoire.

**Table 4:** Conservation of the *F. graminearum* (*Fg*) genes, predicted to encode secreted proteins, among the 57 fungal genomes assessed and the presented according to overall species distribution, host range or lifestyle.

	All secreted proteins	All secreted proteins
Sub-sets	e-5	e-40
**Total gene number**	**574**	
*Fg* specific	**19 (3.31%)**	**103 (17.94%)**
All *Fus*	448 (78.05%)	365 (63.59%)
Unique to all *Fus*	**31 (5.4%)**	**179 (31.19%)**
*Fg +* other non *Fus*	482 (83.97%)	395 (68.82%)
Animal pathogen	**394 (68.64%)**	**289 (50.35%)**
Plant pathogen	460 (80.14%)	372 (64.81%)
Cereal ear pathogen	419 (73%)	337 (58.71%)
Cereal leaf pathogen	437 (76.13%)	344 (59.93%)
Budding yeast	**127 (22.13%)**	**35 (6.1%)**
Biotroph	313 (54.53%)	156 (27.18%)
Hemibiotroph	437 (76.13%)	361 (62.89%)
Necrotroph	422 (73.52%)	330 (57.49%)
Saprotroph	448 (78.05%)	354 (61.67%)

Among the four Fusaria assessed, *F. solani* was the most dissimilar to the *F. graminearum* secretome, showing 82.75% conservation, while *F. oxysporum* f. sp. *lycopersici* and the *F. verticillioides* demonstrated 88.5% and 88% conservation, respectively. The *F. graminearum* secretome was well conserved beyond these four Fusaria, with a total of 83.97% being conserved in at least one additional species. Of the other fungal genomes analysed, the predicted ascomycete secretomes of the rice infecting pathogen *M. oryzae* and the wheat infecting pathogen *S. nodorum* showed the most similarity to the *F. graminearum* secretome, with 66.38% and 66.03% of genes conserved, while only 56.97% of the secretome was conserved in the closely related saprophyte *Trichoderma reesei*. The basidomycete biotroph *U. maydis* demonstrated 38.85% conservation, more than the ascomycete obligate biotroph *B. graminis* f. sp. *hordei* at 30.14%. The oomycete hemibiotroph *P. infestans* demonstrated 32.4% conservation. The yeast secretomes, including the animal pathogen *Candida albicans* at 17.07%, and non-pathogen *Saccharomyces cerevisiae* at 14.81%, were less well conserved.

Conservation of the *F. graminearum* secretome was subsequently determined for the different sub-sets of species depending on host tissue specificity including, animal, plant and cereal ear or cereal leaf infecting pathogens (Table S1). The other Fusaria genomes were excluded from these analyses. Conservation of the *F. graminearum* secretome among 13 animal pathogens was surprisingly high at 68.64%, however this was still lower than the conservation with plant and cereal ear or cereal leaf infecting pathogens at 80.14%, 73% and 76.13%, respectively.

The genomes of plant interacting organisms were divided according to their mode of colonisation. The *F. graminearum* secretome was most well conserved within the nine saprophytic species that obtain nutrition from dead plant material (78.05%). Conservation of the *F. graminearum* secretome among the seven hemibiotrophs and seven necrotrophs was also high at 76.13% and 73.52%, respectively. The only class of plant pathogens within which the *F. graminearum* secretome was poorly conserved was the seven biotrophs (54.53%). This figure is substantially lower than the level of conservation with the 13 animal pathogens. This result we consider being somewhat artifactual and has been caused by the underrepresentation of ascomycete species within the biotroph sub-set. The only genome that is currently available for an ascomycete species that has a biotroph lifestyle is *B. graminis* f. sp. *hordei*. This species forms abundant intracellular haustoria. The other six species of biotrophs were either basidiomycetes or oomycetes, whereas in the animal pathogens examined there were 10 ascomycetes. The two non-pathogens, *S. cerevisiae* and *Schizosaccharomyces pombe*, demonstrated a very poor level of conservation (22.13%) with *F. graminearum*.

When this comparative analysis was repeated using a higher confidence level (p<e^−40^) a similar pattern of conservation and species ranking was revealed (Table 4). However, the number of *F. graminearum* specific, and Fusaria unique, proteins increased substantially. At this p value, 103 genes were still considered to be *F. graminearum* specific and 179 genes were considered to be *Fusarium* specific.

### Comparison of the predicted secretome with published *F. graminearum* proteomic data sets

A proteomic comparison of the secretome from *F. graminearum* grown *in vitro* and *in planta*, identified 122 extracellular proteins and according to Paper and colleagues [58], 68 of these proteins possessed a signal peptide. Only 14 of the 68 proteins identified were detected exclusively *in planta*, and these included a metallopeptidase, a KP4 killer toxin, a pectin lyase and an endoglucanase. A total of 68% of the proteins found in the proteomic study were also identified in our predicted secretome, which was designed to be extra stringent. In the proteomic study, nine proteins detected and found to have a signal peptide, were excluded from the bioinformatically predicted secretome generated by this study due to the stringency of the combined SignalP, TargetP and WolfPSort analysis. The majority of the 68 proteins not detected in the predicted secretome were excluded during the WolfPSort analysis. A cut off score of 18 had been used. For all 68 proteins to have been included a far lower WolfPSort cut off score would have needed to have been used, and this would have raised considerably the potential number of false positives included within these analyses. Even when the WolfPSort score was lowered to 17, this included three proteins where the probability scores from SignalP were only modest. The other 46 proteins detected in the proteomic study, which lacked signal peptides were all excluded from this detailed analysis by the WolfPSort analysis. The authors of the proteomic study [58] concluded that the detection of these proteins may have arisen, because the *Fusarium* cell ruptured during sample preparation. For example, NADP-dependent oxidoreductase and elongation factor 1 are not known in other species to be extracellularly located.

## Discussion

Communication through the secretion of proteins and metabolites frequently defines the outcome of the interaction between a host and a fungal symbiont, irrespective of their lifestyle [7], [59], [60]. During the formation of Fusarium Ear Blight disease an intimate host-pathogen association develops and an extended growth phase occurs in the apoplast, which is extracellular to the living wheat cells [44]. In the original analysis of the newly sequenced *F. graminearum* genome, only TargetP was used to predict the secretome [25]. Since this time, the gene call for *F. graminearum* has been considerably changed through the combined efforts of the BROAD and MIPS. In the current study, a refined *F. graminearum* secretome was predicted by the combination of multiple bioinformatic approaches. This strategy increased the probability of identifying truly secreted proteins. A secretome size of 574 proteins is predicted for *F. graminearum*, representing 4.2% of the predicted total gene repertoire. The cell biology of the different phases of wheat ear infection depicts a situation where *Fusarium* hyphae are exposed to different environments/distinct substrates, thereby causing transcriptional, proteinaceous and metabolic changes. *Fusarium* hyphae in the symptomless phase of infection are in close contact with live plant cells for two to three days [44]. During this prolonged latent period, communication between pathogen and host must occur. After several days the wheat cells die and are intracellularly colonised by the pathogen resulting in the development of visible disease symptoms and asexual sporulation. Transcriptional differences between the two phases of infection have been confirmed for the biosynthetic genes responsible for the virulence factor DON, which showed maximal *TRI* gene expression during symptomless infection [38]. The secretion of DON is hypothesised to inhibit the plant's ability to respond to infection by impeding protein synthesis [61]. Mechanisms in addition to DON mycotoxin may also be required to promote infection, implicating a role for the *F. graminearum* secretome [25]. Therefore, an in depth re-analysis of the secretome's capabilities was undertaken.

The comparative genomics analysis of 57 fungal and oomycete genomes revealed a high level of secretome conservation among filamentous ascomycetes, irrespective of their mode of obtaining nutrition from plant or animal hosts or during a free living lifestyle. This high level of secretome conservation may reflect the ability of *F. graminearum* to survive both as a pathogen and as a saprophyte. The identification of 31 *Fusarium* specific and 25 *F. graminearum* specific secreted proteins, of which all were functionally unannotated proteins, may represent the conserved and unique protein-protein interactions that assist *Fusarium* pathogenicity. The predicted *F. graminearum* secretome, with a size of 1,369 from the initial analysis and 574 from the refined selection (Figure 1) appears larger than the *B. graminis* (248), and *U. maydis* (426) secretomes, but possibly slightly smaller than that of *M. oryzae* (739) [31], [52], [62]. However, the size of these fungal secretomes was predicted using slightly different approaches. Despite representing a large fungal secretome, the refined set of *F. graminearum* secreted proteins demonstrated less species specificity than the biotrophic pathogens *B. graminis* and in particular *U. maydis* where two thirds of the secreted proteins are species specific.

As previously noted, the *F. graminearum* secretome predominantly localises to hot spots of chromosomal recombination and sub-telomeric regions [25] facilitating alterations to the secretome that could enable the pathogen to cope with changes in the host plant response. However, some genes predicted to code for a secreted protein were located in the intervening low or no recombination regions found on each of the four chromosomes. In addition, several small clusters of secreted proteins (ranging from 3–6 genes) were identified in regions of the genome located with either a low or a high level of recombination and in both sub-telomeric and more central locations. Unlike *U. maydis* [52] these *F. graminearum* clusters did not contain genes of similar function and did not represent gene duplication events.

In total 99% of the refined bioinformatic prediction of the *F. graminearum* secretome was supported by transcriptional evidence. During early *F. graminearum* infection *TRI* gene expression is up-regulated at the advancing hyphal front in the florets [63] and the rachis tissue [38]. Along with the array of secreted proteins, DON may inhibit the plant cells ability to detect or respond to infection. The small cysteine-rich secreted proteins, of which many contained internal amino acid repeats, and the additional pathogenicity related protein similar to a circumsporozoite that has been shown to inhibit protein synthesis in cells infected by malaria *Plasmodium* parasites [64] may also play a role in establishing wheat infection. An ability to obtain nutrition from the apoplast and possibly inhibit, or circumvent, plant defences is in agreement with the observed lack of physiological changes to the plant cells during this initial phase of infection [44].

After a latent period of infection wheat host cells die prior to, or at the same time as, *F. graminearum* hyphae penetrate host cells *en masse* [44]. Whether host cell death is induced by the plant in an attempt to limit infection, or by the fungus to obtain nutrition, remains unknown. The two small secreted proteins, related to Snodprot1 from *S. nodorum* that has proven phytotoxin activity [65] also contain the cerato-platanin Pfam07249 domain. In *Ceratocystis fimbriata*, the Snodprot1 protein exists in the fungal cell wall and has been shown to induce host cell phytoalexin synthesis as well as necrosis [66]. In *M. oryzae* the Snodprot1 homologue is required for full virulence [67]. Several *F. graminearum SNODPROT1* homologues were identified in the secretome that have been demonstrated to be transcribed during wheat ear [68]. From the extracellular location, the possible phytotoxic activity of these two small *F. graminearum* secreted proteins may play a role in the induction of host cell death. In wheat, infiltration of high concentrations of DON mycotoxin into healthy leaves has been shown to elicit hydrogen peroxide production and programmed cell death [69]. Therefore, the level or length of exposure to the mycotoxin could also be involved in the induction of host cell death. Interestingly, several lactonohydrolases were predicted in the secretome. A novel lactonohydrolase cloned from *Clonostachys rosea* into *S. pombe* or *Escherichia coli* was able to detoxify the trichothecene mycotoxin, zearalenone [70], [71]. The localised secretion of a lactonohydrolase by *F. graminearum*, may therefore act as a self defence mechanism, in addition to the experimentally proven Tri101 protein [72].

Once within dead plant tissue *F. graminearum* is predicted to secrete an array of PCWDEs and other enzymes, far more than many other fungal pathogens [25], [73]. *F. graminearum* also appears to possess the capacity to utilise the plant cell in its entirety, which is in agreement of the observed phenotype of wheat rachis infection [44]. This ability of the secretome to breakdown the plant cell is probably essential for *F. graminearum* pathogenesis, but will be difficult to test experimentally because of the problem of genetic redundancy. The extensive repertoire of PCWDEs would also assist in the saprophytic phase of the *F. graminearum* lifecycle, which occurs post-harvest [74].

*F. graminearum* may be able to produce a range of antifungal proteins, including FGSG_04745 and four KP4 killer toxins. Their production could prevent additional colonisation by fungal competitors and protect the niche the *F. graminearum* hyphae have occupied. The trichothecene mycotoxins may also have some antifungal activity [75]. The *U. maydis* KP4 killer toxins provide antifungal activity by blocking calcium uptake thereby interfering with calcium signalling [76]. The increased production of antifungal proteins may be essential during late infection, reflecting the vulnerability of the dead plant tissue to further microbial colonisation.

The functional analysis of the secretome revealed the presence of a large set of extracellular proteins with a function in metabolism. This suggests *Fusarium* hyphae can manipulate or directly interfere with the plant's metabolism. Acid and alkaline phosphatases are responsible for protein dephosphorylation, which is pivotal to cell signalling. Alcohol oxidases catalyse the reaction between alcohol and O_2_ releasing an aldehyde and H_2_O_2_, which is an important plant signalling molecule. Salicylate hydroxylase is capable of degrading the plant defence signalling molecule, salicylic acid, which has been shown to be required for maintaining basal defence against *Fusarium* in the floral tissues of Arabidopsis [77], [78] while a delay in salicylic acid signalling has also been associated with increased *Fusarium* susceptibility in wheat ears [79]. The extracellular nucleases indicate the potential to degrade DNA/RNA or interfere with nucleic acid function. Extracellular proteins involved in protein-binding were also highly represented.

Plant PR proteins are rapidly expressed upon the perception of pathogen attack [80]. The secretion by *Fusarium* of related PR proteins, such as PR1 and PR5K is intriguing. The *F. graminearum* PR1-like protein is conserved in *F. oxysporum*, *F. verticillioides*, *F. solani* and *M. oryzae*, while the PR5K-like protein in addition to the aforementioned species is widely conserved in *S. nodorum*, *S. sclerotiorum*, *P. tritici repentis*, *Leptosphaeria maculans*, *T. reesei* and *N. crassa*. The role of PR proteins in fungal pathogenesis has so far not been reported for any interaction.

Approximately half of the predicted secretome encoded for proteins of unknown function (n = 296). These proteins of unknown function could include key effectors that control host species or tissue specificity. To provide some annotation, these sequences were surveyed for functional domains and characteristics for high cysteine content, internal amino acid repeats and the presence of the consensus and/or degenerative RxLR or Y/F/WxC motifs. These additional analyses have provided sequence based annotation for the majority of the predicted secreted proteins of unknown function. A frequent functional domain present in the proteins of unknown function was the NPP1 domain, which has been associated with inducing plant necrosis during *P. infestans* infection [57] and is specifically expressed during the transition between biotrophic-necrotrophic *P. sojae* infection [81]. However, in the *M. graminicola* wheat leaf interaction, which also switches from symptomless to symptomatic infection, the only NPP1 homologue was not required for full virulence [82]. Neither the consensus nor degenerative RxLR motif situated in the N terminus of the predicted proteins was identified in the refined secretome studied here. However, the five secreted protein identified with the YxC motif in close proximity to the signal peptide represents an interesting find that requires further investigation.

This study has greatly increased our understanding of the *F. graminearum* secretome and identified genes coding for secreted proteins that can be considered to be *Fusarium* conserved and *F. graminearum* specific. Once the genomic sequences of additional Fusaria species and strains and other fungal species are published, these secretome predictions can be further refined. In order to achieve a greater understanding of the transcriptional differences between the different phases of *in planta* infection in different plant host species and different tissues, genome wide investigations coupled with a synchronised biological assay that accurately separates the different phases of infection will be required. The use of the *Fusarium* Affymetrix array [83] and/or a next generation deep-RNA sequencing approach would be ideal. The later would also give considerable information, in parallel, on the nature of the induced host responses. The gene models for *F. graminearum* continue to evolve through the increased use of manual sequence corrections [26]. This activity is likely to lead to further refinements to the predicted *F. graminearum* secretome.

## Supporting Information

Table S1 The fungal and oomycete genomes included within the 57 species analysis.
(DOC)

Table S2 The sub-set of *F. graminearum* genes that code for secreted proteins involved in the degradation of the plant cuticle and cell wall, divided according to substrate specificity.
(DOC)

Table S3 The sub-set of *F. graminearum* genes that code for secreted proteins involved in the degradation of the plant cell, divided according to substrate specificity (starch, lipid and protein).
(DOC)

Table S4 The sub-set of *F. graminearum* genes that code for MIPs annotated secreted proteins but not predicted to function in the degradation of plant cells.
(DOC)

Table S5 The sub-set of *F. graminearum* genes that code for secreted proteins with no MIPS annotation, but contain conserved protein functional (Pfam) domains.
(DOC)

Table S6 The sub-set of *F. graminearum* genes that code for cysteine –rich (>5%) unannotated secreted proteins
(DOC)

Spreadsheet S1 (XLS)

## References

[1] Tyler BM (2009). Entering and breaking: virulence effector proteins of oomycete plant pathogens.. Cellular Microbiology.

[2] Bhattacharjee S, Hiller NL, Liolios K, Win J, Kanneganti TD (2006). The malarial host-targeting signal is conserved in the Irish potato famine pathogen.. PLoS Pathogens.

[3] Kale SD, Gu BA, Capelluto DGS, Dou DL, Feldman E (2010). External lipid PI3P mediates entry of eukaryotic pathogen effectors into plant and animal host cells.. Cell.

[4] Deller S, Hammond-Kosack KE, Rudd JJ (2011). The complex interactions between host immunity and non-biotrophic fungal pathogens of wheat leaves.. Journal of Plant Physiology.

[5] Godfrey D, Bohlenius H, Pedersen C, Zhang Z, Emmersen J (2010). Powdery mildew fungal effector candidates share N-terminal Y/F/WxC-motif.. BMC Genomics.

[6] Stergiopoulos I, de Wit PJGM (2009). Fungal Effector Proteins.. Annual Review of Phytopathology.

[7] Tan KC, Oliver RP, Solomon PS, Moffat CS (2010). Proteinaceous necrotrophic effectors in fungal virulence.. Functional Plant Biology.

[8] Ridout CJ, Skamnioti P, Porritt O, Sacristan S, Jones JDG (2006). Multiple avirulence paralogues in cereal powdery mildew fungi may contribute to parasite fitness and defeat of plant resistance.. Plant Cell.

[9] Lievens B, Houterman PM, Rep M (2009). Effector gene screening allows unambiguous identification of *Fusarium oxysporum* f. sp. *lycopersici* races and discrimination from other formae speciales.. FEMS Microbiology Letters.

[10] Valent B, Khang CH (2010). Recent advances in rice blast effector research.. Current Opinion in Plant Biology.

[11] Doehlemann G, van der Linde K, Assmann D, Schwammbach D, Hof A (2009). Pep1, a secreted effector protein of *Ustilago maydis*, is required for successful invasion of plant cells.. PLoS Pathogens.

[12] Marshall R, Kombrink A, Motteram J, Loza-Reyes E, Lucas J (2011). Analysis of two *in planta* expressed LysM effector homologs from the fungus *Mycosphaerella graminicola* reveals novel functional properties and varying contributions to virulence on wheat.. Plant Physiology.

[13] Kloppholz S, Kuhn H, Requena N (2011). A secreted fungal effector of *Glomus intraradices* promotes symbiotic biotrophy.. Current Biology.

[14] van Esse HP, Bolton MD, Stergiopoulos L, de Wit PJGM, Thomma BPHJ (2007). The chitin-binding *Cladosporium fulvum* effector protein Avr4 is a virulence factor.. Molecular Plant-Microbe Interactions.

[15] de Jonge RS, van Esse HP, Kombrink A, Shinya T, Desaki Y (2010). Conserved fungal LysM effector Ecp6 prevents chitin-triggered immunity in plants.. Science.

[16] Verstrepen KJ, Jansen A, Lewitter F, Fink GR (2005). Intragenic tandem repeats generate functional variability.. Nature Genetics.

[17] Levdansky E, Romano J, Shadkchan Y, Sharon H, Verstrepen KJ (2007). Coding tandem repeats generate diversity in *Aspergillus fumigatus* genes.. Eukaryotic Cell.

[18] Rudd JJ, Antoniw J, Marshall R, Motteram J, Fraaije B (2010). Identification and characterisation of *Mycosphaerella graminicola* secreted or surface-associated proteins with variable intragenic coding repeats.. Fungal Genetics and Biology.

[19] Khang CH, Berruyer R, Giraldo MC, Kankanala P, Park SY (2010). Translocation of *Magnaporthe oryzae* effectors into rice cells and their subsequent cell-to-cell movement.. Plant Cell.

[20] Skibbe DS, Doehlemann G, Fernandes J, Walbot V (2010). Maize tumors caused by *Ustilago maydis* require organ-specific genes in host and pathogen.. Science.

[21] Dublin HJ, Glichrist L, Reeves J, McNab A (1997). Fusarium Head Scab: Global Status and Prospects.

[22] Kimura M, Tokai T, Takahashi-Ando N, Ohsato S, Fujimura M (2007). Molecular and genetic studies of *Fusarium* trichothecene biosynthesis: Pathways, genes, and evolution.. Bioscience Biotechnology and Biochemistry.

[23] Hook S, Williams R, Edwards C, Dodgson G (2007). Guidelines to minimise risk of fusarium mycotoxins in cereals.

[24] Gale LR, Bryant JD, Calvo S, Giese H, Katan T (2005). Chromosome complement of the fungal plant pathogen *Fusarium graminearum* based on genetic and physical mapping and cytological observations.. Genetics.

[25] Cuomo CA, Gueldener U, Xu JR, Trail F, Turgeon BG (2007). The *Fusarium graminearum* genome reveals a link between localized polymorphism and pathogen specialization.. Science.

[26] Wong P, Walter M, Lee W, Mannhaupt G, Munsterkotter M (2011). FGDB: revisiting the genome annotation of the plant pathogen *Fusarium graminearum*.. Nucleic Acids Research.

[27] Beacham A, Antoniw J, Hammond-Kosack KE (2009). A genomic fungal foray.. Biologist.

[28] Beacham A (2011). Pathogenicity of *Fusarium graminearum* and *Fusarium culmorum* on wheat ears.

[29] Xu JR (2000). MAP kinases in fungal pathogens.. Fungal Genetics and Biology.

[30] Haas BJ, Kamoun S, Zody MC, Jiang RHY, Handsaker RE (2009). Genome sequence and analysis of the Irish potato famine pathogen *Phytophthora infestans*.. Nature.

[31] Spanu PD, Abbott JC, Amselem J, Burgis TA, Soanes DM (2010). Genome expansion and gene loss in powdery mildew fungi reveal tradeoffs in extreme parasitism.. Science.

[32] Proctor RH, Hohn TM, McCormick SP, Desjardins AE (1995). *Tri6* encodes an unusual zinc-finger protein involved in regulation of trichothecene biosynthesis in *Fusarium sporotrichioides*.. Applied and Environmental Microbiology.

[33] Proctor RH, Hohn TM, McCormick SP (1995). Reduced virulence of *Gibberella zeae* caused by disruption of a trichothecene toxin synthetic gene.. Molecular Plant-Microbe Interactions.

[34] Cuzick A, Urban M, Hammond-Kosack K (2008). *Fusarium graminearum* gene deletion mutants *map1* and *tri5* reveal similarities and differences in the pathogenicity requirements to cause disease on Arabidopsis and wheat floral tissue.. New Phytologist.

[35] Harris LJ, Desjardins AE, Plattner RD, Nicholson P, Butler G (1999). Possible role of trichothecene mycotoxins in virulence of *Fusarium graminearum* on maize.. Plant Disease.

[36] Maier FJ, Miedaner T, Hadeler B, Felk A, Salomon S (2006). Involvement of trichothecenes in fusarioses of wheat, barley and maize evaluated by gene disruption of the trichodiene synthase *(Tri5)* gene in three field isolates of different chemotype and virulence.. Molecular Plant Pathology.

[37] Kimura M, Kaneko I, Komiyama M, Takatsuki A, Koshino H (1998). Trichothecene 3-O-acetyltransferase protects both the producing organism and transformed yeast from related mycotoxins - Cloning and characterization of Tri101.. Journal of Biological Chemistry.

[38] Brown NA, Bass C, Baldwin TK, Chen H, Massot F (2011). Characterisation of the *Fusarium graminearum*-wheat floral interaction.. Journal of Pathogens.

[39] Jansen C, von Wettstein D, Schafer W, Kogel KH, Felk A (2005). Infection patterns in barley and wheat spikes inoculated with wild-type and trichodiene synthase gene disrupted *Fusarium graminearum*.. Proceedings of the National Academy of Sciences of the United States of America.

[40] Dorman CJ, Corcoran CP (2009). Bacterial DNA topology and infectious disease.. Nucleic Acids Research.

[41] O Croinin T, Carroll RK, Kelly A, Dorman CJ (2006). Roles for DNA supercoiling and the Fis protein in modulating expression of virulence genes during intracellular growth of *Salmonella enterica* serovar *typhimurium*.. Molecular Microbiology.

[42] Baldwin TK, Urban M, Brown NA, Hammond-Kosack KE (2010). A role for topoisomerase I in *Fusarium graminearum* and *F. culmorum* pathogenesis and sporulation.. Molecular Plant-Microbe Interactions.

[43] Voigt CA, Schäfer W, Salomon S (2005). A secreted lipase of *Fusarium graminearum* is a virulence factor required for infection of cereals.. Plant Journal.

[44] Brown NA, Urban M, van de Meene AML, Hammond-Kosack KE (2010). The infection biology of *Fusarium graminearum*: Defining the pathways of spikelet to spikelet colonisation in wheat ears.. Fungal Biology.

[45] Muller O, Schreier PH, Uhrig JF (2008). Identification and characterization of secreted and pathogenesis-related proteins in *Ustilago maydis*.. Molecular Genetics and Genomics.

[46] Emanuelsson O, Brunak S, von Heijne G, Nielsen H (2007). Locating proteins in the cell using TargetP, SignalP and related tools.. Nature Protocols.

[47] Emanuelsson O, Nielsen H, Brunak S, von Heijne G (2000). Predicting subcellular localization of proteins based on their N-terminal amino acid sequence.. Journal of Molecular Biology.

[48] Eisenhaber B, Schneider G, Wildpaner M, Eisenhaber F (2004). A sensitive predictor for potential GPI lipid modification sites in fungal protein sequences and its application to genome-wide studies for *Aspergillus nidulans, Candida albicans Neurospora crassa, Saccharomyces cerevisiae* and *Schizosaccharomyces pombe*.. Journal of Molecular Biology.

[49] Horton P, Park KJ, Obayashi T, Nakai K (2006). Protein subcellular localization prediction with WOLF PSORT.. Proceedings of the 4th Asia-Pacific Bioinformatics Conference.

[50] Heger A, Holm L (2000). Rapid automatic detection and alignment of repeats in protein sequences.. Proteins-Structure Function and Genetics.

[51] Antoniw J, Beacham AM, Baldwin TK, Urban M, Rudd JJ (2011). OmniMapFree: a unified tool to visualise and explore sequenced genomes.. BMC Bioinformatics.

[52] Kamper J, Kahmann R, Bolker M, Ma LJ, Brefort T (2006). Insights from the genome of the biotrophic fungal plant pathogen *Ustilago maydis*.. Nature.

[53] Hane JK, Lowe RGT, Solomon PS, Tan KC, Schoch CL (2007). Dothideomycete-plant interactions illuminated by genome sequencing and EST analysis of the wheat pathogen *Stagonospora nodorum*.. Plant Cell.

[54] Koltin Y, Day PR (1976). Inheritance of killer phenotypes and double stranded RNA in *Ustilago maydis*.. Proceedings of the National Academy of Sciences of the United States of America.

[55] Xue CY, Park G, Choi WB, Zheng L, Dean RA (2002). Two novel fungal virulence genes specifically expressed in appressoria of the rice blast fungus.. Plant Cell.

[56] McCormick SP, Alexander NJ (2002). *Fusarium* Tri8 encodes a trichothecene C-3 esterase.. Applied and Environmental Microbiology.

[57] Fellbrich G, Romanski A, Varet A, Blume B, Brunner F (2002). NPP1, a *Phytophthora*-associated trigger of plant defense in parsley and *Arabidopsis*.. Plant Journal.

[58] Paper JM, Scott-Craig JS, Adhikari ND, Cuomo CA, Walton JD (2007). Comparative proteomics of extracellular proteins *in vitro* and *in planta* from the pathogenic fungus *Fusarium graminearum*.. Proteomics.

[59] Gan PHP, Rafiqi M, Hardham AR, Dodds PN (2010). Effectors of biotrophic fungal plant pathogens.. Functional Plant Biology.

[60] Howlett BJ (2006). Secondary metabolite toxins and nutrition of plant pathogenic fungi.. Current Opinion in Plant Biology.

[61] Ueno Y (1984). Toxicological features of T-2 toxin and related trichothecenes.. Fundamental and Applied Toxicology.

[62] Dean RA, Talbot NJ, Ebbole DJ, Farman ML, Mitchell TK (2005). The genome sequence of the rice blast fungus *Magnaporthe grisea*.. Nature.

[63] Boenisch MJ, Schäfer W (2011). *Fusarium graminearum* forms mycotoxin producing infection structures on wheat (*Triticum aestivum L*.).. BMC Plant Biology.

[64] Menard R, Sultan AA, Cortes C, Altszuler R, vanDijk MR (1997). Circumsporozoite protein is required for development of malaria sporozoites in mosquitoes.. Nature.

[65] Hall N, Keon JPR, Hargreaves JA (1999). A homologue of a gene implicated in the virulence of human fungal diseases is present in a plant fungal pathogen and is expressed during infection.. Physiological and Molecular Plant Pathology.

[66] Pazzagli L, Cappugi G, Manao G, Camici G, Santini A (1999). Purification, characterization, and amino acid sequence of cerato-platanin, a new phytotoxic protein from *Ceratocystis fimbriata* f. sp *platani*.. Journal of Biological Chemistry.

[67] Jeong JS, Mitchell TK, Dean RA (2007). The *Magnaporthe grisea* snodprot1 homolog, MSP1, is required for virulence.. FEMS Microbiology Letters.

[68] Lysoe E, Seong KY, Kistler HC (2011). The transcriptome of *Fusarium graminearum* during the infection of wheat.. Molecular Plant-Microbe Interactions.

[69] Desmond OJ, Manners JM, Stephens AE, Maclean DJ, Schenk PM (2008). The Fusarium mycotoxin deoxynivalenol elicits hydrogen peroxide production, programmed cell death and defence responses in wheat.. Molecular Plant Pathology.

[70] Takahashi-Ando N, Kimura M, Kakeya H, Osada H, Yamaguchi I (2002). A novel lactonohydrolase responsible for the detoxification of zearalenone: enzyme purification and gene cloning.. Biochemical Journal.

[71] Takahashi-Ando N, Ohsato S, Shibata T, Hamamoto H, Yamaguchi I (2004). Metabolism of zearalenone by genetically modified organisms expressing the detoxification gene from *Clonostachys rosea*.. Applied and Environmental Microbiology.

[72] McCormick SP, Alexander NJ, Trapp SE, Hohn TM (1999). Disruption of *TRI101*, the gene encoding trichothecene 3-O-acetyltransferase, from *Fusarium sporotrichioides*.. Applied and Environmental Microbiology.

[73] Soanes DM, Richards TA, Talbot NJ (2007). Insights from sequencing fungal and oomycete genomes: What can we learn about plant disease and the evolution of pathogenicity?. Plant Cell.

[74] Parry DW, Jenkinson P, McLeod L (1995). Fusarium ear blight (scab) in small grain cereals - a review.. Plant Pathology.

[75] Lutz MP, Feichtinger G, Defago G, Duffy B (2003). Mycotoxigenic Fusarium and deoxynivalenol production repress chitinase gene expression in the biocontrol agent *Trichoderma atroviride* P1.. Applied and Environmental Microbiology.

[76] Gage MJ, Bruenn J, Fischer M, Sanders D, Smith TJ (2001). KP4 fungal toxin inhibits growth in *Ustilago maydis* by blocking calcium uptake.. Molecular Microbiology.

[77] Cuzick A, Lee S, Gezan S, Hammond-Kosack KE (2008). NPR1 and EDS11 contribute to host resistance against *Fusarium culmorum* in Arabidopsis buds and flowers.. Molecular Plant Pathology.

[78] Makandar R, Nalam V, Chaturvedi R, Jeannotte R, Sparks AA (2010). Involvement of salicylate and jasmonate signaling pathways in Arabidopsis interaction with *Fusarium graminearum*.. Molecular Plant-Microbe Interactions.

[79] Ding LN, Xu HB, Yi HY, Yang LM, Kong ZX (2011). Resistance to hemi-biotrophic *F. graminearum* infection is associated with coordinated and ordered expression of diverse defense signaling pathways.. PLoS ONE.

[80] Sels J, Mathys J, De Coninck BMA, Cammue BPA, De Bolle MFC (2008). Plant pathogenesis-related (PR) proteins: A focus on PR peptides.. Plant Physiology and Biochemistry.

[81] Qutob D, Kamoun S, Gijzen M (2002). Expression of a *Phytophthora sojae* necrosis-inducing protein occurs during transition from biotrophy to necrotrophy.. The Plant Journal.

[82] Motteram J, Kufner I, Deller S, Brunner F, Hammond-Kosack KE (2009). Molecular characterization and functional analysis of *MgNLP*, the sole NPP1 domain-containing protein, from the fungal wheat leaf pathogen *Mycosphaerella graminicola*.. Molecular Plant-Microbe Interaction.

[83] Gueldener U, Seong KY, Boddu J, Cho S, Trail F (2006). Development of a *Fusarium graminearum* Affymetrix GeneChip for profiling fungal gene expression *in vitro* and in *planta*.. Fungal Genet Biol.

