# Supplementary material for: The Predicted Secretome of the Plant Pathogenic Fungus Fusarium graminearum: A Refined Comparative Analysis
Source: PLoS One. 2012 Apr 6;7(4):e33731. doi: 10.1371/journal.pone.0033731 (PMC3320895; doi:10.1371/journal.pone.0033731)
Supplement: Table S1 — The fungal and oomycete genomes included within the 57 species analysis. (DOC) [file pone.0033731.s001.doc]

**Supplementary table S1 The fungal and oomycete genomes included within the 57 species analysis. The genomes screened for the presence of *F. graminearum* secretome genes, included A) plant pathogens, B) saprophytes, C) animal pathogens and D) non-pathogens. Conservation data was generated according to sub-sets relevant to host / tissue specificity and pathogenic lifestyle.**

| **Analysis** | **Fungal / Oomycete genomes** | **Host** | | **Cereal Path** | | **Pathogenicv lifestyle** | | **Organ Specificity** | | |
| --- | --- | --- | --- | --- | --- | --- | --- | --- | --- | --- |
| *A* | *Fusarium graminearum* | P, ICA | | C, N | | S, N | | F, Se, St, E | | |
|  | *Fusarium oxysporum* | P, ICA | | C, N | | S, N | | R, St | | |
|  | *Fusarium solani* | P, ICA | | C, N | | S, N | | R | | |
|  | *Fusarium verticillioides* | P, ICA | | C, N | | S, N | | Se, St, E, R | | |
|  | *Alternaria brassicicola* | P | | C, N | | N | | L, Se, St, P | | |
|  | *Blumeria graminis^h^* | P | | C | | B | | L | | |
|  | *Botrytis cinerea* | P | | N | | N | | F | | |
|  | *Cochliobolus heterotrophus* | P | | C | | N | | L | | |
|  | *Hyaloperonospora arabidopsis^oh^* | P | | N | | B | | L | | |
|  | *Hyaloperonospora parasitica^oh^* | P | | N | | B | | L | | |
|  | *Leptosphaeria maculans* | P | | N | | H | | St, L | | |
|  | *Magnaporthe grisea* | P | | C | | H | | L, E | | |
|  | *Melampsora laricis-populina^b^* | P | | N | | B | | L | | |
|  | *Mycosphaerella fijiensis* | P | | N | | H | | L | | |
|  | *Mycosphaerella graminicola* | P | | C | | H | | L | | |
|  | *Phytophthora infestans^o^* | P | | N | | H | | T | | |
|  | *Phytophthora ramorum^o^* | P | | N | | H | | L, St | | |
|  | *Phytophthora sojae^o^* | P | | N | | H | | R, St | | |
|  | *Puccinia graminis^bh^* | P | | C | | B | | L, St | | |
|  | *Pyrenophora tritici repens* | P | | C | | N | | L, St, Se, E | | |
|  | *Sclerotinia sclerotiorum* | P | | N | | N | | L, F, St, P | | |
|  | *Sporisorium reilianum^b^* | P | | C | | B | | E, Se | | |
|  | *Stagonospora nodorum* | P | | C | | N | | L, E | | |
|  | *Ustilago maydis^b^* | P | | C | | B | | E, Se St | | |
|  | *Verticillium dahliae* | P | | N | | N | | R, St, L | | |
| *B* | *Neurospora crassa* | S | | - | | S | | - | | |
|  | *Phanerochaete chrysosporium^b^* | S | | - | | S | | - | | |
|  | *Trichoderma reesei* | S | | - | | S | | - | | |
|  | *Trichoderma virens* | S | | - | | S | | - | | |
|  | *Aspergillus nidulans* | S, ICA | | - | | S | | - | | |
|  | *Chaetomium globosum* | S, ICA | | - | | S | | - | | |
|  | *Rhizopus oryzae^m^* | S, ICA | | - | | S | | - | | |
| **Analysis** | **Fungal genomes** | | **Host** | | **Cereal Path** | | **Pathogenic lifestyle** | | **Organ Specificity** |  |
| *C* | *Aspergillus fumigatus* | | S, ICA | | - | | OP | | - |  |
|  | *Candida albicans* | | ICA | | - | | OP | | - |  |
|  | *Candida glabrata* | | ICA | | - | | OP | | - |  |
|  | *Coccidioides immitis* | | ICA | | - | | OP | | - |  |
|  | *Cryptococcus neoformans^b^* | | ICA | | - | | OP | | - |  |
|  | *Histoplasma capsulatum* | | A, ICA | | - | | OP | | - |  |
|  | *Batrachochytrium dendrobatidis^r^* | | A | | - | | P | | - |  |
|  | *Blastomyces dermatitidis* | | A | | - | | OP | | - |  |
|  | *Microsporum gypseum* | | A | | - | | OP | | - |  |
|  | *Paracoccidioides brasiliensis* | | A | | - | | OP | | - |  |
| *D* | *Saccharomyces cerevisiae* | | N | | - | | S | | - |  |
|  | *Schizosaccharomyces pombe* | | N | | - | | S | | - |  |

All organisms are fungal ascomycetes unless labelled otherwise.

Phyla: b = basidiomycetes, o = oomycetes. Order: m = mucoraceae, r = rhizophydiales

Host: A = animal, ICA = immunocompromised animal, P = plant, S = saprophyte

Cereal Path: C = pathogen of cereal host, N = pathogen of non-cereal host

Plant pathogenic lifestyle: B = biotroph, H = hemibiotroph, N = necrotroph, S = saprophyte, OP = opportunistic pathogen, P = pathogen

Organ specificity: E = ear, F = flower, L = leaf, Se= seed, St = stem, P = petal, R = root

- = not applicable
