# Supplementary material for: The Predicted Secretome of the Plant Pathogenic Fungus Fusarium graminearum: A Refined Comparative Analysis
Source: PLoS One. 2012 Apr 6;7(4):e33731. doi: 10.1371/journal.pone.0033731 (PMC3320895; doi:10.1371/journal.pone.0033731)
Supplement: Table S2 — The sub-set of F. graminearum genes that code for secreted proteins involved in the degradation of the plant cuticle and cell wall, divided according to substrate specificity. (DOC) [file pone.0033731.s002.doc]

**Supplementary table S2** The sub-set of *F. graminearum* genes that code for secreted proteins involved in the degradation of the plant cuticle and cell wall, divided according to substrate specificity. EC numbers were obtained from http://mips.helmholtz-muenchen.de/genre/proj/FGDB/

| | **Locus ID** | **Cell wall component** | **Mode of action** | **MIPS annotation** | **EC** | | --- | --- | --- | --- | --- | | FGSG_01621 | Cellulose | hydrolysis, hydrolysis of O-glycosyl bond | probable cellulase precursor | 3.2.1.4 / 3.2.1.6 | | FGSG_02202 | Cellulose | hydrolysis of O-glycosyl bond | probable endoglucanase IV precursor | 3.2.1.4 | | FGSG_02658 | Cellulose | hydrolysis of O-glycosyl bond | probable endoglucanase | 3.2.1.4 | | FGSG_03143 | Cellulose |  | related to glycosyl hydrolase |  | | FGSG_03632 | Cellulose |  | related to cellulose binding protein CEL1 |  | | FGSG_03695 | Cellulose | hydrolysis of O-glycosyl bond | related to endoglucanase IV precursor | 3.2.1.4 | | FGSG_03795 | Cellulose | hydrolysis, hydrolysis of O-glycosyl bond | probable cellulase precursor | 3.2.1.4 / 3.2.1.6 | | FGSG_04681 | Cellulose | hydrolysis of O-glycosyl bond | probable endoglucanase IV precursor | 3.2.1.4 | | FGSG_05851 | Cellulose | hydrolysis of O-glycosyl bond, transglycosylation | related to endoglucanase I precursor | 3.2.1.4 / 3.2.1.151 | | FGSG_06110 | Cellulose | hydrolysis of O-glycosyl bond | probable endoglucanase IV precursor | 3.2.1.4 | | FGSG_06278 | Cellulose | hydrolysis of O-glycosyl bond | probable glucan 1,4-alpha-glucosidase | 3.2.1.1 / 3.2.1.3 | | FGSG_07944 | Cellulose |  | related to glucanase | 3.2.1.58 | | FGSG_08011 | Cellulose |  | related to cellulose binding protein CEL1 |  | | FGSG_11037 | Cellulose | hydrolysis of O-glycosyl bond | probable endoglucanase I precursor | 3.2.1.4 | | FGSG_11326 | Cellulose | hydrolysis of O-glycosyl bond | probable glucan 1,4-alpha-glucosidase | 3.2.1.1 / 3.2.1.3 | | FGSG_11488 | Cellulose |  | related to cellulose binding protein CEL1 |  | | FGSG_12160 | Cellulose |  | probable cel1 protein precursor |  | | FGSG_13245 | Cellulose | hydrolysis of O-glycosyl bond | probable endoglucanase IV | 3.2.1.4 | |  |  |  |  |  | | FGSG_01685 | Cellulose / Cellobiose | hydrolysis of O-glycosyl bond | related to beta-glucosidase | 3.2.1.21 | | FGSG_03387 | Cellulose / Cellobiose | hydrolysis of O-glycosyl bond | probable beta-glucosidase precursor | 3.2.1.21 | | FGSG_03570 | Cellulose / Cellobiose | hydrolysis of O-glycosyl bond | probable beta-glucosidase 1 precursor | 3.2.1.21 | | FGSG_03628 | Cellulose / Cellobiose | hydrolysis of O-glycosyl bond | probable cellulose 1,4-beta-cellobiosidase II precursor | 3.2.1.91 | | FGSG_03858 | Cellulose / Cellobiose | hydrolysis of O-glycosyl bond | probable beta-glucosidase precursor | 3.2.1.21 | | **Locus ID** | **Cell wall component** | **Mode of action** | **MIPS annotation** | **EC** | | FGSG_04953 | Cellulose / Cellobiose | hydrolysis of O-glycosyl bond | probable beta-glucosidase precursor | 3.2.1.21 | | FGSG_06605 | Cellulose / Cellobiose | hydrolysis of O-glycosyl bond | probable beta-glucosidase | 3.2.1.21 | | FGSG_10615 | Cellulose / Cellobiose |  | related to beta-glucosidase precursor |  | | FGSG_11559 | Cellulose / Cellobiose | hydrolysis of O-glycosyl bond | related to beta-glucosidase 1 precursor | 3.2.1.21 | |  |  |  |  |  | | FGSG_02917 | Cellobiose | oxidation, redox reaction, reduction | related to cellobiose dehydrogenase | 1.1.3.25 / 1.1.99.18 | | FGSG_05983 | Cellobiose | oxidation, redox reaction, reduction | related to cellobiose dehydrogenase | 1.1.3.25 / 1.1.99.18 | | FGSG_09085 | Cellobiose | oxidation, redox reaction, reduction | probable cellobiose dehydrogenase | 1.1.3.25 / 1.1.99.18 | |  |  |  |  |  | | FGSG_00783 | Hemicellulose | hydrolysis of carboxylic ester | related to acetylxylan esterase precursor | 3.1.1.74 | | FGSG_00989 | Hemicellulose |  | probable rhamnogalacturonase B precursor |  | | FGSG_01803 | Hemicellulose | hydrolysis of O-glycosyl bond | probable alpha-galactosidase C precursor | 3.2.1.22 | | FGSG_02059 | Hemicellulose | hydrolysis of O-glycosyl bond | related to alpha-galactosidase precursor | 3.2.1.22 | | FGSG_02314 | Hemicellulose | hydrolysis of O-glycosyl bond | related to beta-mannosidase | 1.1.1.49 / 3.2.1.25 / 3.2.1.132 | | FGSG_03002 | Hemicellulose | hydrolysis of O-glycosyl bond | related to arabinan endo-1,5-alpha-L-arabinosidase A precursor | 3.2.1.99 | | FGSG_03003 | Hemicellulose |  | related to alpha-N-arabinofuranosidase / alpha-L-arabinofuranosidase |  | | FGSG_03049 | Hemicellulose | hydrolysis of O-glycosyl bond | related to alpha-L-arabinofuranosidase II precursor | 3.2.1.55 | | FGSG_03194 | Hemicellulose | hydrolysis of O-glycosyl bond | probable endopolygalacturonase | 3.2.1.15 | | FGSG_03343 | Hemicellulose | hydrolysis of O-glycosyl bond | related to beta-galactosidase | 3.2.1.23 | | FGSG_03384 | Hemicellulose | hydrolysis of O-glycosyl bond | probable exopolygalacturonase | 3.2.1.67 / 3.2.1.82 | | FGSG_03569 | Hemicellulose | oxidation, redox reaction, reduction | probable galactose oxidase | 1.1.3.9 | | FGSG_03624 | Hemicellulose | hydrolysis, hydrolysis of O-glycosyl bond | probable endo-1,4-beta-xylanase A precursor | 3.2.1.8 | | FGSG_03629 | Hemicellulose | hydrolysis, hydrolysis of O-glycosyl bond | probable alpha-glucuronidase precursor | 3.2.1.131 / 3.2.1.139 | | FGSG_03813 | Hemicellulose | hydrolysis of O-glycosyl bond | probable alpha-L-arabinofuranosidase | 3.2.1.37 / 3.2.1.55 | | FGSG_03867 | Hemicellulose | hydrolysis of carboxylic ester | probable acetylxylan esterase | 3.1.1.1 / 3.1.1.72 / 3.1.1.73 | | FGSG_03904 | Hemicellulose | hydrolysis of O-glycosyl bond | probable beta-galactosidase | 3.2.1.23 | | FGSG_03905 | Hemicellulose |  | related to putative arabinase |  | | **Locus ID** | **Cell wall component** | **Mode of action** | **MIPS annotation** | **EC** | | FGSG_03922 | Hemicellulose | hydrolysis of O-glycosyl bond | related to class I alpha-mannosidase 1B | 3.2.1.113 | | FGSG_04678 | Hemicellulose | hydrolysis of O-glycosyl bond | related to beta-mannanase | 3.2.1.78 | | FGSG_04689 | Hemicellulose | hydrolysis of O-glycosyl bond | probable rhamnogalacturonase A precursor | 3.2.1.15 | | FGSG_04848 | Hemicellulose |  | probable rhamnogalacturonan acetylesterase precursor |  | | FGSG_06117 | Hemicellulose |  | related to rhamnogalacturonate lyase precursor |  | | FGSG_06463 | Hemicellulose | hydrolysis of O-glycosyl bond | related to alpha-L-arabinofuranosidase A precursor | 3.2.1.55 | | FGSG_07625 | Hemicellulose | hydrolysis of O-glycosyl bond | probable alpha-L-arabinofuranosidase precursor | 3.2.1.8 / 3.2.1.55 | | FGSG_07639 | Hemicellulose | hydrolysis of O-glycosyl bond | related to xylosidase/arabinosidase | 3.2.1.37 / 3.2.1.55 | | FGSG_07993 | Hemicellulose | hydrolysis of O-glycosyl bond | related to xylan 1,4-beta-xylosidase | 3.2.1.37 | | FGSG_09093 | Hemicellulose | oxidation, redox reaction, reduction | related to galactose oxidase precursor | 1.1.3.9 | | FGSG_10670 | Hemicellulose | hydrolysis of carboxylic ester | probable acetylxylan esterase precursor | 3.1.1.72 | | FGSG_10999 | Hemicellulose | hydrolysis, hydrolysis of O-glycosyl bond | endo-1,4-beta-xylanase | 3.2.1.8 | | FGSG_11011 | Hemicellulose | hydrolysis of O-glycosyl bond | probable PGU1 - Endo-polygalacturonase | 3.2.1.15 | | FGSG_11032 | Hemicellulose |  | galactose oxidase precursor [GAO] | 1.1.3.9 | | FGSG_11048 | Hemicellulose | hydrolysis of O-glycosyl bond | probable arabinogalactan endo-1,4-beta-galactosidase | 3.2.1.89 | | FGSG_11066 | Hemicellulose | hydrolysis of O-glycosyl bond | related to beta-mannanase | 3.2.1.68 / 3.2.1.78 | | FGSG_11169 | Hemicellulose | hydrolysis of O-glycosyl bond | related to alpha-galactosidase precursor | 3.2.1.22 | | FGSG_11229 | Hemicellulose |  | related to acetylxylan esterase |  | | FGSG_11304 | Hemicellulose | hydrolysis, hydrolysis of O-glycosyl bond | related to endo-1,4-beta-xylanase | 3.2.1.8 / 3.2.1.4 | | FGSG_11487 | Hemicellulose | hydrolysis, hydrolysis of O-glycosyl bond | related to endo-1,4-beta-xylanase | 3.2.1.8 | | FGSG_11548 | Hemicellulose | hydrolysis of carboxylic ester | related to acetylxylan esterase precursor | 3.1.1.72 / 3.1.1.74 | | FGSG_12047 | Hemicellulose |  | probable endo-1,4-beta-xylanase A precursor |  | | FGSG_13189 | Hemicellulose | hydrolysis, hydrolysis of O-glycosyl bond | probable endo-1,4-beta-xylanase | 3.2.1.8 | |  |  |  |  |  | |  |  |  |  |  | |  |  |  |  |  | |  |  |  |  |  | | **Locus ID** | **Cell component** | **Mode of action** | **MIPS annotation** | **EC** | | FGSG_02987 | Hemicellulose / Lignin | carboxylic ester hydrolysis | related to feruloyl esterase B precursor | 3.1.1.20 / 3.1.1.72 / 3.1.1.73 / 3.5.1.11 | | FGSG_03217 | Hemicellulose / Lignin | carboxylic ester hydrolysis | related to feruloyl esterase B precursor | 3.1.1.20 / 3.1.1.73 / 3.5.1.11 | | FGSG_11428 | Hemicellulose / Lignin | carboxylic ester hydrolysis | probable feruloyl esterase B precursor | 3.1.1.1 / 3.1.1.72 / 3.1.1.73 | | FGSG_12548 | Hemicellulose / Lignin | carboxylic ester hydrolysis | related to feruloyl esterase B precursor | 3.1.1.20 / 3.1.1.72 / 3.1.1.73 | |  |  |  |  |  | | FGSG_01607 | Pectin | beta-elimination, elimination, ester hydrolysis | probable pectin lyase precursor | 4.2.2.10 | | FGSG_02977 | Pectin | elimination | probable pectate lyase | 4.2.2.2 | | FGSG_03121 | Pectin | beta-elimination, elimination, ester hydrolysis | probable pectin lyase precursor | 4.2.2.10 | | FGSG_03131 | Pectin | elimination | related to pectate lyase L precursor | 4.2.2.2 / 4.2.2.9 | | FGSG_03406 | Pectin | hydrolysis of carboxylic ester | probable pectinesterase precursor | 3.1.1.11 | | FGSG_03908 | Pectin | elimination | probable pectate lyase 1 | 4.2.2.2 / 4.2.99.3 | | FGSG_04439 | Pectin | hydrolysis of carboxylic ester | related to pectinesterase | 3.1.1.11 | | FGSG_04864 | Pectin | elimination | probable pectate lyase | 4.2.2.2 | | FGSG_07794 | Pectin | elimination | probable pectate lyase 1 | 4.2.2.2 / 4.2.99.3 | | FGSG_09291 | Pectin | elimination | probable pectate lyase 1 | 4.2.2.2 / 4.2.99.3 | | FGSG_11094 | Pectin | elimination | probable pectate lyase | 4.2.2.2 | | FGSG_11163 | Pectin | elimination | probable pectate lyase 1 | 4.2.2.2 / 4.2.99.3 | |  |  |  |  |  | | FGSG_02330 | Lignin | oxidation, redox reaction, reduction | related to laccase precursor | 1.10.3.3 | | FGSG_03507 | Lignin | oxidation, redox reaction, reduction | related to laccase precursor | 1.10.3.2 | | FGSG_09646 | Lignin | oxidation, redox reaction, reduction | related to laccase precursor | 1.10.3.2 | | FGSG_04434 | Lignin |  | related to peroxidase | 1.11.1.11 / 1.11.1.13 | | FGSG_03436 | Lignin | oxidation, redox reaction, reduction | related to chloroperoxidase | 1.11.1.10 | |  |  |  |  |  | |  |  |  |  |  | |  |  |  |  |  | |  |  |  |  |  | | **Locus ID** | **Cell component** | **Mode of action** | **MIPS annotation** | **EC** | | FGSG_02651 | Callose | hydrolysis of O-glycosyl bond | related to endo-1,3-beta-glucanase | 3.2.1.6 | | FGSG_03529 | Callose | hydrolysis of O- and S- glycosyl bond | related to glucan 1,3-beta-glucosidase | 3.2.1.58 | | FGSG_03827 | Callose | hydrolysis of O-glycosyl bond | related to endo-1,3-beta-glucanase | 3.2.1.6 | | FGSG_04768 | Callose | hydrolysis of O-glycosyl bond | related to endo-1,3-beta-glucanase | 3.2.1.6 | | FGSG_06616 | Callose | hydrolysis of O- and S- glycosyl bond | probable beta-1,3 exoglucanase precursor | 3.2.1.58 | | FGSG_07238 | Callose | hydrolysis of O- and S- glycosyl bond | related to beta-1,3 exoglucanase | 3.2.1.58 | | FGSG_08265 | Callose | hydrolysis of O- and S- glycosyl bond | related to SPR1 - exo-1,3-beta-glucanase precursor | 3.2.1.75 | | FGSG_09445 | Callose | hydrolysis of O- and S- glycosyl bond | related to beta-1,3 exoglucanase precursor | 3.2.1.58 | | FGSG_11006 | Callose | hydrolysis of O-and S- glycosyl bond, transglycosylation | probable glucan endo-1,3-beta-glucosidase bgn13.1 precursor | 3.2.1.39 / 3.2.1.58 | |  |  |  |  | | FGSG_02890 | Cuticle | carboxylic ester hydrolysis | probable cutinase precursor | 3.1.1.74 | | FGSG_03457 | Cuticle | carboxylic ester hydrolysis | probable cutinase 1 precursor | 3.1.1.74 | |
| --- | --- | --- | --- | --- | --- | --- | --- | --- | --- | --- | --- | --- | --- | --- | --- | --- | --- | --- | --- | --- | --- | --- | --- | --- | --- | --- | --- | --- | --- | --- | --- | --- | --- | --- | --- | --- | --- | --- | --- | --- | --- | --- | --- | --- | --- | --- | --- | --- | --- | --- | --- | --- | --- | --- | --- | --- | --- | --- | --- | --- | --- | --- | --- | --- | --- | --- | --- | --- | --- | --- | --- | --- | --- | --- | --- | --- | --- | --- | --- | --- | --- | --- | --- | --- | --- | --- | --- | --- | --- | --- | --- | --- | --- | --- | --- | --- | --- | --- | --- | --- | --- | --- | --- | --- | --- | --- | --- | --- | --- | --- | --- | --- | --- | --- | --- | --- | --- | --- | --- | --- | --- | --- | --- | --- | --- | --- | --- | --- | --- | --- | --- | --- | --- | --- | --- | --- | --- | --- | --- | --- | --- | --- | --- | --- | --- | --- | --- | --- | --- | --- | --- | --- | --- | --- | --- | --- | --- | --- | --- | --- | --- | --- | --- | --- | --- | --- | --- | --- | --- | --- | --- | --- | --- | --- | --- | --- | --- | --- | --- | --- | --- | --- | --- | --- | --- | --- | --- | --- | --- | --- | --- | --- | --- | --- | --- | --- | --- | --- | --- | --- | --- | --- | --- | --- | --- | --- | --- | --- | --- | --- | --- | --- | --- | --- | --- | --- | --- | --- | --- | --- | --- | --- | --- | --- | --- | --- | --- | --- | --- | --- | --- | --- | --- | --- | --- | --- | --- | --- | --- | --- | --- | --- | --- | --- | --- | --- | --- | --- | --- | --- | --- | --- | --- | --- | --- | --- | --- | --- | --- | --- | --- | --- | --- | --- | --- | --- | --- | --- | --- | --- | --- | --- | --- | --- | --- | --- | --- | --- | --- | --- | --- | --- | --- | --- | --- | --- | --- | --- | --- | --- | --- | --- | --- | --- | --- | --- | --- | --- | --- | --- | --- | --- | --- | --- | --- | --- | --- | --- | --- | --- | --- | --- | --- | --- | --- | --- | --- | --- | --- | --- | --- | --- | --- | --- | --- | --- | --- | --- | --- | --- | --- | --- | --- | --- | --- | --- | --- | --- | --- | --- | --- | --- | --- | --- | --- | --- | --- | --- | --- | --- | --- | --- | --- | --- | --- | --- | --- | --- | --- | --- | --- | --- | --- | --- | --- | --- | --- | --- | --- | --- | --- | --- | --- | --- | --- | --- | --- | --- | --- | --- | --- | --- | --- | --- | --- | --- | --- | --- | --- | --- | --- | --- | --- | --- | --- | --- | --- | --- | --- | --- | --- | --- | --- | --- | --- | --- | --- | --- | --- | --- | --- | --- | --- | --- | --- | --- | --- | --- | --- | --- | --- | --- | --- | --- | --- | --- | --- | --- | --- | --- | --- | --- | --- | --- | --- | --- | --- | --- | --- | --- | --- | --- | --- | --- | --- | --- | --- | --- | --- | --- | --- | --- | --- | --- | --- | --- | --- | --- | --- | --- | --- | --- | --- | --- | --- | --- | --- | --- | --- | --- | --- | --- | --- | --- | --- | --- | --- | --- | --- | --- | --- | --- | --- | --- | --- | --- | --- | --- | --- | --- | --- | --- | --- | --- | --- | --- | --- | --- | --- | --- | --- | --- | --- | --- | --- | --- | --- | --- | --- | --- | --- | --- | --- | --- | --- | --- | --- | --- | --- | --- | --- | --- | --- | --- | --- | --- | --- | --- | --- | --- | --- | --- | --- | --- | --- | --- | --- | --- | --- | --- | --- | --- | --- | --- | --- | --- | --- | --- | --- | --- | --- | --- | --- | --- | --- | --- | --- | --- | --- | --- | --- | --- | --- | --- | --- | --- | --- | --- | --- | --- | --- | --- | --- | --- | --- | --- | --- | --- | --- | --- | --- | --- | --- | --- | --- | --- | --- | --- | --- | --- | --- | --- | --- | --- | --- | --- | --- | --- | --- | --- | --- | --- | --- | --- | --- | --- | --- | --- | --- |
