# Supplementary material for: The Predicted Secretome of the Plant Pathogenic Fungus Fusarium graminearum: A Refined Comparative Analysis
Source: PLoS One. 2012 Apr 6;7(4):e33731. doi: 10.1371/journal.pone.0033731 (PMC3320895; doi:10.1371/journal.pone.0033731)
Supplement: Table S3 — The sub-set of F. graminearum genes that code for secreted proteins involved in the degradation of the plant cell, divided according to substrate specificity (starch, lipid and protein). (DOC) [file pone.0033731.s003.doc]

**Supplementary table S3** The sub-set of *F. graminearum* genes that code for secreted proteins involved in the degradation of the plant cell, divided according to substrate specificity (starch, lipid and protein). EC numbers were obtained from http://mips.helmholtz-muenchen.de/genre/proj/FGDB/

| | **Locus ID** | **Cell component** | **Mode of action** | **MIPS annotation** | **EC** | | --- | --- | --- | --- | --- | | FGSG_03842 | Starch | Hydrolysis of O-glycosyl bond | related to alpha-amylase A precursor | 3.2.1.1 | | FGSG_04704 | Starch |  | related to glucoamylase precursor |  | | FGSG_03034 | Starch | Oxidation, reduction | related to glucose dehydrogenase | 1.1.3.7 / 1.1.99.1 | |  |  |  |  |  | | FGSG_01240 | Lipid | Hydrolysis, hydrolysis of carboxylic ester, acetylation | related to triacylglycerol lipase | 3.1.1.3 | | FGSG_01603 | Lipid | Hydrolysis, hydrolysis of carboxylic ester, acetylation | probable triacylglycerol lipase V precursor | 3.1.1.13 / 3.1.1.3 | | FGSG_03012 | Lipid |  | related to triacylglycerol lipase V precursor |  | | FGSG_03095 | Lipid |  | related to triacylglycerol lipase V precursor |  | | FGSG_03209 | Lipid | Hydrolysis, hydrolysis of carboxylic ester, acetylation | related to triacylglycerol lipase II precursor | 3.1.1.3 / 3.1.1.7 | | FGSG_03243 | Lipid | Hydrolysis, hydrolysis of carboxylic ester, acetylation | related to triacylglycerol lipase II precursor | 3.1.1.3 | | FGSG_03583 | Lipid | Hydrolysis, hydrolysis of carboxylic ester, acetylation | related to triacylglycerol lipase V precursor | 3.1.1.3 / 3.1.1.42 / 3.1.1.8 | | FGSG_03687 | Lipid | Hydrolysis, hydrolysis of carboxylic ester, acetylation | related to triacylglycerol lipase V precursor | 3.1.1.3 | | FGSG_03846 | Lipid | Hydrolysis, hydrolysis of carboxylic ester, acetylation | related to lipase 1 | 3.1.1.3 | | FGSG_04818 | Lipid | Hydrolysis, hydrolysis of carboxylic ester, acetylation | related to triacylglycerol lipase precursor | 3.1.1.3 / 3.1.1.73 | | FGSG_05906 | Lipid | Hydrolysis, hydrolysis of carboxylic ester, acetylation | probable triacylglycerol lipase precursor | 3.1.1.3 | | FGSG_06437 | Lipid | Hydrolysis, hydrolysis of carboxylic ester, acetylation | related to triacylglycerol lipase V precursor | 3.1.1.3 | | FGSG_09099 | Lipid | Hydrolysis, hydrolysis of carboxylic ester, acetylation | related to triacylglycerol lipase V precursor | 3.1.1.8 | | FGSG_09181 | Lipid | Hydrolysis, hydrolysis of carboxylic ester, acetylation | related to triacylglycerol lipase II precursor | 3.1.1.3 | | FGSG_10713 | Lipid | Hydrolysis, hydrolysis of carboxylic ester, acetylation | related to triacylglycerol lipase V precursor | 3.1.1.3 | | FGSG_11112 | Lipid | Hydrolysis, hydrolysis of carboxylic ester, acetylation | related to lipase/acylhydrolase | 3.2.1.4 / 3.1.1.72 | | FGSG_11227 | Lipid | Hydrolysis, hydrolysis of carboxylic ester, acetylation | related to lipase B precursor | 3.1.1.3 | | FGSG_11386 | Lipid | Hydrolysis, hydrolysis of carboxylic ester, acetylation | related to triacylglycerol lipase II precursor | 3.1.1.1 | | FGSG_11036 | Lipid | Hydrolysis of carboxylic ester | related to esterase D | 3.1.1.72 | | **Locus ID** | **Cell component** | **Mode of action** | **MIPS annotation** | **EC** | | FGSG_02918 | Protein | Hydrolysis of peptide bond | related to aspartic proteinase, pepstatin-sensitive | 3.4.23.1 / 3.4.23.15 / 3.4.23.24 | | FGSG_04397 | Protein | Hydrolysis of peptide bond | related to aspartic proteinase, pepstatin-sensitive | 3.4.23.1 / 3.4.23.5 | | FGSG_04817 | Protein |  | related to serine protease |  | | FGSG_06332 | Protein |  | related to subtilisin-like serine protease |  | | FGSG_06895 | Protein | Hydrolysis of peptide bond | probable PRC1 - carboxypeptidase y, serine-type protease | 3.4.16.5 | | FGSG_07775 | Protein | Hydrolysis of proteins, including elastin, by preferential cleavage | probable aspartic proteinase precursor | 3.4.23.18 / 3.4.23.20 / 3.4.23.22 | | FGSG_08464 | Protein | Hydrolysis of proteins with broad specificity | related to alkaline protease (oryzin) | 3.4.21.14 / 3.4.21.62 / 3.4.21.63 | | FGSG_08583 | Protein |  | related to aspartyl proteinase SAP3 precursor |  | | FGSG_09382 | Protein | Hydrolysis of proteins with broad specificity | probable alkaline protease (oryzin) | 3.4.21.14 / 3.4.21.62 / 3.4.21.63 | | FGSG_10525 | Protein | Hydrolysis of proteins with broad specificity | related to subtilisin-like serine protease | 3.4.21.14 / 3.4.21.62 / 3.4.21.63 | | FGSG_10595 | Protein | Hydrolysis of proteins with broad specificity | related to alkaline protease (oryzin) | 3.4.21.14 / 3.4.21.62 / 3.4.21.63 | | FGSG_10712 | Protein | Hydrolysis of proteins with broad specificity | related to alkaline protease (oryzin) | 3.4.21.14 / 3.4.21.62 / 3.4.21.63 | | FGSG_12544 | Protein |  | related to proteinase R precursor |  | | FGSG_00806 | Protein | Hydrolysis of peptide bond | probable endopeptidase K | 3.4.21.14 / 3.4.21.62 / 3.4.21.63 | | FGSG_02976 | Protein | Hydrolysis of peptide bond | probable endopeptidase K | 3.4.21.64 | | FGSG_03315 | Protein | Hydrolysis of peptide bond | related to endopeptidase K | 3.4.21.14 / 3.4.21.62 / 3.4.21.63 | | FGSG_03975 | Protein |  | related to aspartic-type signal peptidase |  | | FGSG_04546 | Protein | Hydrolysis of peptide bond | related to serine-type carboxypeptidase f precursor | 3.4.16.6 | | FGSG_05797 | Protein | Hydrolysis of peptide bond | probable Serine-type carboxypeptidase F precursor | 3.4.16.6 | | FGSG_12142 | Protein | Hydrolysis of peptide bond, release of N-terminal residue from tripeptide | related to tripeptidyl-peptidase I | 3.4.11.4 / 3.4.14.9 | | FGSG_11280 | Protein | Hydrolysis of carboxylic ester, transesterification | probable acetylesterase | 3.1.1.11 | | FGSG_02015 | Protein | Acetylation, carboxylic ester hydrolysis, transesterification | related to esterase | 3.1.1.3 / 3.1.1.42 / 3.1.1.8 | | FGSG_03331 | Protein | Hydrolysis of peptide bond | related to YSP3 - subtilisin-like protease III | 3.4.21.48 | | FGSG_03432 | Protein | Hydrolysis of peptide bond | probable endothiapepsin precursor | 3.4.23.18 / 3.4.23.20 / 3.4.23.22 | | FGSG_03467 | Protein |  | probable extracellular elastinolytic metalloproteinase precursor |  | |  |  |  |  |  | | **Locus ID** | **Cell component** | **Mode of action** | **MIPS annotation** | **EC** | | FGSG_03769 | Protein | Hydrolysis of peptide bond | related to carboxypeptidase | 3.4.16.5 / 3.4.16.6 | | FGSG_04097 | Protein |  | related to PRC1 - carboxypeptidase y, serine-type protease |  | | FGSG_04527 | Protein | Hydrolysis of peptide bond | related to carboxypeptidase | 3.4.16.5 / 3.4.16.6 | | FGSG_05245 | Protein | Hydrolysis of peptide bond | related to aminopeptidase | 3.4.11.10 | | FGSG_06572 | Protein |  | probable subtilisin-like serine protease |  | | FGSG_08196 | Protein | Hydrolysis of peptide bond | related to aspergillopepsin II precursor | 3.4.23.19 / 3.4.23.32 | | FGSG_10982 | Protein | Hydrolysis of peptide bond | related to dipeptidyl aminopeptidase B | 3.4.14.5 | | FGSG_11249 | Protein | Hydrolysis of peptide bond | related to carboxypeptidase A | 3.4.17.1 / 3.4.17.15 | | FGSG_11472 | Protein |  | probable subtilisin-like serine protease |  | | FGSG_00028 | Protein |  | probable metalloprotease MEP1 |  | | FGSG_08289 | Protein |  | related to neutral proteinase |  | | FGSG_08454 | Protein | Hydrolysis of peptide bond | related to carboxypeptidase | 3.4.16.5 / 3.4.16.6 | |  |  |  |  |  | |
| --- | --- | --- | --- | --- | --- | --- | --- | --- | --- | --- | --- | --- | --- | --- | --- | --- | --- | --- | --- | --- | --- | --- | --- | --- | --- | --- | --- | --- | --- | --- | --- | --- | --- | --- | --- | --- | --- | --- | --- | --- | --- | --- | --- | --- | --- | --- | --- | --- | --- | --- | --- | --- | --- | --- | --- | --- | --- | --- | --- | --- | --- | --- | --- | --- | --- | --- | --- | --- | --- | --- | --- | --- | --- | --- | --- | --- | --- | --- | --- | --- | --- | --- | --- | --- | --- | --- | --- | --- | --- | --- | --- | --- | --- | --- | --- | --- | --- | --- | --- | --- | --- | --- | --- | --- | --- | --- | --- | --- | --- | --- | --- | --- | --- | --- | --- | --- | --- | --- | --- | --- | --- | --- | --- | --- | --- | --- | --- | --- | --- | --- | --- | --- | --- | --- | --- | --- | --- | --- | --- | --- | --- | --- | --- | --- | --- | --- | --- | --- | --- | --- | --- | --- | --- | --- | --- | --- | --- | --- | --- | --- | --- | --- | --- | --- | --- | --- | --- | --- | --- | --- | --- | --- | --- | --- | --- | --- | --- | --- | --- | --- | --- | --- | --- | --- | --- | --- | --- | --- | --- | --- | --- | --- | --- | --- | --- | --- | --- | --- | --- | --- | --- | --- | --- | --- | --- | --- | --- | --- | --- | --- | --- | --- | --- | --- | --- | --- | --- | --- | --- | --- | --- | --- | --- | --- | --- | --- | --- | --- | --- | --- | --- | --- | --- | --- | --- | --- | --- | --- | --- | --- | --- | --- | --- | --- | --- | --- | --- | --- | --- | --- | --- | --- | --- | --- | --- | --- | --- | --- | --- | --- | --- | --- | --- | --- | --- | --- | --- | --- | --- | --- | --- | --- | --- | --- | --- | --- | --- | --- | --- | --- | --- | --- | --- | --- | --- | --- | --- | --- | --- | --- | --- | --- | --- | --- | --- | --- | --- | --- | --- | --- | --- | --- | --- | --- | --- | --- | --- | --- | --- | --- | --- | --- | --- | --- | --- | --- | --- | --- | --- | --- | --- | --- | --- | --- | --- |
