# Supplementary material for: The Predicted Secretome of the Plant Pathogenic Fungus Fusarium graminearum: A Refined Comparative Analysis
Source: PLoS One. 2012 Apr 6;7(4):e33731. doi: 10.1371/journal.pone.0033731 (PMC3320895; doi:10.1371/journal.pone.0033731)
Supplement: Table S4 — The sub-set of F. graminearum genes that code for MIPs annotated secreted proteins but not predicted to function in the degradation of plant cells. (DOC) [file pone.0033731.s004.doc]

**Supplementary table S4** The sub-set of *F. graminearum* genes that code for MIPs annotated secreted proteins but not predicted to function in the degradation of plant cells.

| **FGSG_ID** |  | **MIPs Annotation** |
| --- | --- | --- |
| FGSG_00006 |  | related to gEgh 16 protein |
| FGSG_00023 |  | probable spherulin 1A precursor |
| FGSG_00060 |  | related to KP4 killer toxin |
| FGSG_00061 |  | related to KP4 killer toxin |
| FGSG_00062 |  | related to KP4 killer toxin |
| FGSG_00100 |  | related to 6-hydroxy-d-nicotine oxidase |
| FGSG_00642 |  | related to spore coat protein SP96 precursor |
| FGSG_00742 |  | related to S-adenosylmethionine:diacylglycerol 3-amino-3-carboxypropyl transferase |
| FGSG_01595 |  | putative protein [EST hit] |
| FGSG_01660 |  | putative protein [EST hit] |
| FGSG_01763 |  | putative protein [EST hit] |
| FGSG_01818 |  | related to bacterial leucyl aminopeptidase |
| FGSG_01829 |  | related to aldose 1-epimerase |
| FGSG_01831 |  | related to trihydrophobin precursor |
| FGSG_01988 |  | related to monophenol monooxygenase (tyrosinase) |
| FGSG_02269 |  | putative protein [EST hit] |
| FGSG_02339 |  | related to SUC2 - invertase (sucrose hydrolyzing enzyme) |
| FGSG_02422 |  | related to spore coat protein SP96 precursor |
| FGSG_02686 |  | related to ribonucleases |
| FGSG_02893 |  | related to protein-arginine deiminase type II |
| FGSG_03109 |  | related to plant PR-1 class of pathogen related proteins |
| FGSG_03307 |  | related to isoamyl alcohol oxidase |
| FGSG_03379 |  | related to ribonucleases |
| FGSG_03526 |  | OrfE - unknown, trichothecene gene cluster |
| FGSG_03531 |  | monooxygenase |
| FGSG_03532 |  | trichothecene 3-O-esterase |
| FGSG_03585 |  | putative protein [EST hit] |
| FGSG_03616 |  | related to isoamyl alcohol oxidase |
| FGSG_03816 |  | probable lactonohydrolase |
| FGSG_03865 |  | related to L-sorbosone dehydrogenase |
| FGSG_03916 |  | related to Rds1 protein |
|  |  |  |
| **FGSG_ID** |  | **MIPs Annotation** |
| FGSG_03954 |  | related to S.fumigata Asp FII |
| FGSG_03972 |  | related to isoamyl alcohol oxidase |
| FGSG_04504 |  | related to acid phosphatase precursor |
| FGSG_04732 |  | related to 6-hydroxy-d-nicotine oxidase |
| FGSG_04745 |  | related to antifungal protein |
| FGSG_04980 |  | related to palmitoyl-(protein) hydrolase |
| FGSG_05163 |  | probable heterokaryon incompatibility Het-C protein |
| FGSG_05757 |  | probable rAsp f 9 allergen |
| FGSG_05763 |  | related to glyoxal oxidase precursor |
| FGSG_05933 |  | related to acid phosphatase precursor |
| FGSG_06087 |  | related to spore coat protein SP96 precursor |
| FGSG_06438 |  | related to isoamyl alcohol oxidase |
| FGSG_06451 |  | related to levanase |
| FGSG_06465 |  | related to haloacetate dehalogenase H-1 |
| FGSG_06610 |  | related to alkaline phosphatase D precursor |
| FGSG_06612 |  | related to oxalate decarboxylase |
| FGSG_06733 |  | probable catalase-3 |
| FGSG_07569 |  | related to RF2 protein |
| FGSG_07608 |  | related to acid phosphatase precursor |
| FGSG_07661 |  | related to 6-hydroxy-d-nicotine oxidase |
| FGSG_07678 |  | related to acid phosphatase Pho610 |
| FGSG_07691 |  | related to OrfH, unknown gene in trichothecene gene cluster |
| FGSG_07721 |  | probable arylsulfatase |
| FGSG_07838 |  | probable isoamyl alcohol oxidase |
| FGSG_07934 |  | putative protein [EST hit] |
| FGSG_08007 |  | related to monophenol monooxygenase (tyrosinase) |
| FGSG_08116 |  | related to salicylate hydroxylase |
| FGSG_08150 |  | related to PLB1 - phospholipase B (lysophospholipase) |
| FGSG_08549 |  | related to pathogenesis-related protein PR5K (thaumatin family) |
| FGSG_08824 |  | related to berberine bridge enzyme |
| FGSG_09353 |  | related to gEgh 16 protein |
| FGSG_09358 |  | related to phosphatidylcholine-sterol acyltransferase precursor |
| FGSG_09390 |  | related to circumsporozoite protein precursor |
| FGSG_09586 |  | probable phosphatidylglycerol/phosphatidylinositol transfer protein |
| FGSG_10206 |  | putative protein [EST hit] |
|  |  |  |
| **FGSG_ID** |  | **MIPs Annotation** |
| FGSG_10212 |  | probable SnodProt1 PRECURSOR |
| FGSG_10495 |  | putative protein [EST hit] |
| FGSG_10561 |  | related to RF2 protein |
| FGSG_10587 |  | related to peroxisomal amine oxidase (copper-containing) |
| FGSG_10609 |  | related to 6-hydroxy-d-nicotine oxidase |
| FGSG_10611 |  | related to 6-hydroxy-d-nicotine oxidase |
| FGSG_10656 |  | related to OrfH - unknown, trichothecene gene cluster |
| FGSG_10675 |  | related to lactonohydrolase |
| FGSG_10677 |  | related to peroxisomal amine oxidase (copper-containing) |
| FGSG_10986 |  | related to alcohol oxidase |
| FGSG_10998 |  | related to 6-hydroxy-D-nicotine oxidase |
| FGSG_11095 |  | related to carbonic anhydrase |
| FGSG_11097 |  | related to glyoxal oxidase precursor |
| FGSG_11106 |  | related to monophenol monooxygenase (tyrosinase) |
| FGSG_11164 |  | probable trypsin precursor |
| FGSG_11190 |  | probable ribonuclease T1 |
| FGSG_11205 |  | probable SnodProt1 precursor |
| FGSG_11236 |  | related to non-hemolytic phospholipase C precursor |
| FGSG_11318 |  | related to RF2 protein |
| FGSG_11517 |  | related to monophenol monooxygenase (tyrosinase) |
| FGSG_11528 |  | related to monophenol monooxygenase (tyrosinase) |
| FGSG_11602 |  | probable brefeldin A resistance protein |
| FGSG_11645 |  | related to proteoglycan |
| FGSG_11745 |  | related to putative venom metalloproteinase jararhagin precursor |
| FGSG_12127 |  | probable isoamyl alcohol oxidase |
| FGSG_12251 |  | probable arylsulfatase |
| FGSG_12256 |  | related to 6-hydroxy-d-nicotine oxidase |
| FGSG_12369 |  | probable catalase 2 |
| FGSG_12390 |  | related to alcohol oxidase |
| FGSG_12414 |  | related to conidiation-specific protein CON-13 |
| FGSG_12492 |  | related to cell wall mannoprotein |
| FGSG_13450 |  | related to isoamyl alcohol oxidase |
| FGSG_13505 |  | putative protein [EST hit] |
| FGSG_13840 |  | probable isoamyl alcohol oxidase |
| FGSG_13992 |  | related to peroxisomal short-chain alcohol dehydrogenase |
| FGSG_15003 |  | related to dnase1 protein |
