# Supplementary material for: The Predicted Secretome of the Plant Pathogenic Fungus Fusarium graminearum: A Refined Comparative Analysis
Source: PLoS One. 2012 Apr 6;7(4):e33731. doi: 10.1371/journal.pone.0033731 (PMC3320895; doi:10.1371/journal.pone.0033731)
Supplement: Table S5 — The sub-set of F. graminearum genes that code for secreted proteins with no MIPS annotation, but contain conserved protein functional (Pfam) domains. (DOC) [file pone.0033731.s005.doc]

**Supplementary table S5** The sub-set of *F. graminearum* genes that code for secreted proteins with no MIPS annotation, but contain conserved protein functional (Pfam) domains.

| **FGSG-ID** | **Pfam domains** |
| --- | --- |
| FGSG_10500 | pfam00070,pfam07992 |
| FGSG_02551 | pfam00141 |
| FGSG_03050 | pfam00144 |
| FGSG_04656 | pfam00144 |
| FGSG_07996 | pfam00144 |
| FGSG_08136 | pfam00144 |
| FGSG_09109 | pfam00150 |
| FGSG_02354 | pfam00187,pfam00704 |
| FGSG_00569 | pfam00188 |
| FGSG_08415 | pfam00251,pfam08244 |
| FGSG_03348 | pfam00264 |
| FGSG_03901 | pfam00561 |
| FGSG_12067 | pfam00561,pfam08386 |
| FGSG_02360 | pfam00657 |
| FGSG_03129 | pfam00657 |
| FGSG_03612 | pfam00657 |
| FGSG_12119 | pfam00657 |
| FGSG_13883 | pfam00728 |
| FGSG_01982 | pfam00775 |
| FGSG_04685 | pfam00775 |
| FGSG_11232 | pfam00890,pfam01266,pfam01593,pfam03486,pfam07992 |
| FGSG_12206 | pfam00890,pfam03486 |
| FGSG_10435 | pfam01034,pfam01822,pfam03154,pfam03935,pfam03999,pfam04415,pfam04484,pfam04683,pfam05109,pfam05110,pfam05539,pfam05642,pfam05792,pfam05955,pfam06075,pfam06933,pfam07010,pfam07218,pfam07263,pfam08550,pfam08580,pfam08601,pfam08639,pfam08702,pfam08729,pfam09319,pfam09595,pfam09726,pfam09786,pfam10033 |
| FGSG_08958 | pfam01048 |
| FGSG_03304 | pfam01083 |
| FGSG_01570 | pfam01083,pfam04683,pfam05642 |
| FGSG_03960 | pfam01185 |
| FGSG_03708 | pfam01328 |
| FGSG_02228 | pfam01425 |
| FGSG_09046 | pfam01425 |
| FGSG_05052 | pfam01546,pfam07687 |
| FGSG_02263 | pfam01547 |
| FGSG_08825 | pfam01738 |
| FGSG_03574 | pfam01822 |
| FGSG_03365 | pfam03372 |
| FGSG_03986 | pfam03372 |
| FGSG_09475 | pfam03372 |
| FGSG_06443 | pfam03403 |
| **FGSG-ID** | **Pfam domains** |
| FGSG_00294 | pfam03663 |
| FGSG_03609 | pfam04616 |
| FGSG_07207 | pfam04616 |
| FGSG_07695 | pfam04616 |
| FGSG_08041 | pfam04616 |
| FGSG_11366 | pfam04616 |
| FGSG_00031 | pfam05109 |
| FGSG_02448 | pfam05109 |
| FGSG_02888 | pfam05109 |
| FGSG_15123 | pfam05498 |
| FGSG_04743 | pfam05592 |
| FGSG_11170 | pfam05592 |
| FGSG_03394 | pfam05630 |
| FGSG_06017 | pfam05630 |
| FGSG_11493 | pfam05630 |
| FGSG_10676 | pfam05792,pfam10528 |
| FGSG_03521 | pfam06172 |
| FGSG_11206 | pfam06742,pfam06863 |
| FGSG_08978 | pfam07174 |
| FGSG_08115 | pfam07632 |
| FGSG_12551 | pfam07944 |
| FGSG_07556 | pfam07992 |
| FGSG_03724 | pfam08386 |
| FGSG_01688 | pfam08881 |
| FGSG_10622 | pfam08881 |
| FGSG_10551 | pfam09044 |
| FGSG_03911 | pfam09056 |
| FGSG_06993 | pfam09352 |
| FGSG_01728 | pfam09362 |
| FGSG_06775 | pfam09362 |
| FGSG_01588 | pfam09770 |
| FGSG_04824 | pfam09770 |
| FGSG_11348 | pfam10282 |
| FGSG_04739 | pfam10528 |
| FGSG_04858 | pfam10528 |
