# Supplementary material for: The Predicted Secretome of the Plant Pathogenic Fungus Fusarium graminearum: A Refined Comparative Analysis
Source: PLoS One. 2012 Apr 6;7(4):e33731. doi: 10.1371/journal.pone.0033731 (PMC3320895; doi:10.1371/journal.pone.0033731)
Supplement: Table S6 — The sub-set of F. graminearum genes that code for cysteine –rich (>5%) unannotated secreted proteins (DOC) [file pone.0033731.s006.doc]

**Supplementary table S6 The sub-set of *F. graminearum* genes that code for cysteine –rich (>5 %) unannotated secreted proteins.**

| **FGSG_ID** | **Mature peptide length** | **Number of cysteines** | **Percentage of cysteines in peptide** | **RADAR repeats** | **Pfam domains** |
| --- | --- | --- | --- | --- | --- |
| FGSG_00002 | 714 | 70 | 9.8 | 15 | - |
| FGSG_08978 | 199 | 12 | 6.03 | 2 | pfam07174 |
| FGSG_07899 | 197 | 10 | 5.08 | - | - |
| FGSG_15251 | 47 | 6 | 12.77 | - | - |
| FGSG_09132 | 189 | 10 | 5.29 | 2 | - |
| FGSG_03599 | 77 | 10 | 12.99 | 3 | - |
| FGSG_00847 | 48 | 4 | 8.33 | - | - |
| FGSG_15437 | 53 | 8 | 15.09 | - | - |
| FGSG_15123 | 59 | 4 | 6.78 | - | pfam05498 |
| FGSG_00260 | 60 | 6 | 10 | - | - |
| FGSG_10554 | 180 | 9 | 5 | 2 | - |
| FGSG_09066 | 64 | 8 | 12.5 | 2 | - |
| FGSG_01688 | 222 | 12 | 5.41 | 2 | pfam08881 |
| FGSG_15448 | 71 | 8 | 11.27 | - | - |
| FGSG_01754 | 212 | 14 | 6.6 | 2 | - |
| FGSG_01776 | 212 | 14 | 6.6 | 2 | - |
| FGSG_15142 | 71 | 8 | 11.27 | - | - |
| FGSG_15469 | 74 | 6 | 8.11 | 2 | - |
| FGSG_06712 | 129 | 16 | 12.4 | 3 | - |
| FGSG_04239 | 186 | 14 | 7.53 | 3 | - |
| FGSG_00114 | 82 | 6 | 7.32 | - | - |
| FGSG_07755 | 86 | 6 | 6.98 | - | - |
| FGSG_02898 | 1195 | 98 | 8.2 | 10 | - |
| FGSG_05841 | 89 | 8 | 8.99 | - | - |
| FGSG_07684 | 217 | 16 | 7.37 | 3 | - |
| FGSG_11047 | 91 | 8 | 8.79 | 2 | - |
| FGSG_11225 | 94 | 8 | 8.51 | 2 | - |
| FGSG_08090 | 95 | 6 | 6.32 | - | - |
| FGSG_09127 | 97 | 8 | 8.25 | - | - |
| FGSG_04740 | 239 | 18 | 7.53 | 3 | - |
| FGSG_02378 | 99 | 8 | 8.08 | - | - |
| FGSG_02674 | 100 | 6 | 6 | 2 | - |
| FGSG_11647 | 100 | 8 | 8 | - | - |
| FGSG_00029 | 158 | 11 | 6.96 | - | - |
| FGSG_03820 | 102 | 6 | 5.88 | 2 | - |
| FGSG_08987 | 103 | 6 | 5.83 | - | - |
| FGSG_05609 | 103 | 6 | 5.83 | 2 | - |
| FGSG_08180 | 103 | 6 | 5.83 | 2 | - |
| FGSG_03969 | 482 | 58 | 12.03 | 12 | - |
| **FGSG_ID** | **Mature peptide length** | **Number of cysteines** | **Percentage of cysteines in peptide** | **RADAR repeats** | **Pfam domains** |
| FGSG_03960 | 153 | 9 | 5.88 | 2 | pfam01185 |
| FGSG_04429 | 974 | 52 | 5.34 | 6 | - |
| FGSG_10592 | 106 | 6 | 5.66 | - | - |
| FGSG_00230 | 108 | 6 | 5.56 | - | - |
| FGSG_05046 | 112 | 8 | 7.14 | - | - |
| FGSG_00129 | 149 | 9 | 6.04 | 2 | - |
| FGSG_03130 | 120 | 6 | 5 | 2 | - |
| FGSG_12644 | 140 | 7 | 5 | 3 | - |
| FGSG_02685 | 128 | 8 | 6.25 | 2 | - |
| FGSG_02309 | 130 | 8 | 6.15 | - | - |
| FGSG_08210 | 133 | 10 | 7.52 | 2 | - |
| FGSG_03334 | 132 | 10 | 7.58 | 2 | - |
| FGSG_07871 | 236 | 17 | 7.2 | 2 | - |
| FGSG_11156 | 330 | 19 | 5.76 | 4 | - |
| FGSG_12504 | 101 | 7 | 6.93 | - | - |
| FGSG_12554 | 101 | 7 | 6.93 | - | - |
| FGSG_12214 | 79 | 10 | 12.66 | 2 | - |
| FGSG_12439 | 638 | 58 | 9.09 | 12 | - |
| FGSG_10622 | 98 | 5 | 5.1 | - | pfam08881 |
| FGSG_12300 | 89 | 5 | 5.62 | 2 | - |
| FGSG_15260 | 69 | 5 | 7.25 | 2 | - |
| FGSG_15661 | 77 | 10 | 12.99 | - | - |
